# IMPACT is a GCN2 inhibitor that limits lifespan in *Caenorhabditis elegans*

**DOI:** 10.1186/s12915-016-0301-2

**Published:** 2016-10-07

**Authors:** Rafael C. Ferraz, Henrique Camara, Evandro A. De-Souza, Silas Pinto, Ana Paula F. Pinca, Richard C. Silva, Vitor N. Sato, Beatriz A. Castilho, Marcelo A. Mori

**Affiliations:** 1 Department of Biophysics, Escola Paulista de Medicina, Universidade Federal de São Paulo, São Paulo, Brazil; 2 Department of Biochemistry and Tissue Biology, Universidade Estadual de Campinas, Campinas, Brazil; 3 Department of Microbiology, Immunology and Parasitology, Escola Paulista de Medicina, Universidade Federal de São Paulo, São Paulo, Brazil

**Keywords:** IMPACT, GCN2, Dietary restriction, Integrated stress response, Aging

## Abstract

**Background:**

The General Control Nonderepressible 2 (GCN2) kinase is a conserved member of the integrated stress response (ISR) pathway that represses protein translation and helps cells to adapt to conditions of nutrient shortage. As such, GCN2 is required for longevity and stress resistance induced by dietary restriction (DR). IMPACT is an ancient protein that inhibits GCN2.

**Results:**

Here, we tested whether IMPACT down-regulation mimics the effects of DR in *C. elegans*. Knockdown of the *C. elegans IMPACT* homolog *impt-1* activated the ISR pathway and increased lifespan and stress resistance of worms in a *gcn-2*-dependent manner. *Impt-1* knockdown exacerbated DR-induced longevity and required several DR-activated transcription factors to extend lifespan, among them SKN-1 and DAF-16, which were induced during larval development and adulthood, respectively, in response to *impt-1* RNAi.

**Conclusions:**

IMPACT inhibits the ISR pathway, thus limiting the activation of stress response factors that are beneficial during aging and required under DR.

**Electronic supplementary material:**

The online version of this article (doi:10.1186/s12915-016-0301-2) contains supplementary material, which is available to authorized users.

## Background

Overall decreases in fertility and mortality rates have aged the world’s population [1]. Generalized and progressive tissue deterioration with aging is associated with impaired stress responses and results in increased risk of chronic diseases [2]. The increasing number of elderly people has therefore raised public health concerns and highlighted the necessity of interventions to attenuate age-related dysfunctions.

Dietary restriction (DR) is one of the best characterized strategies that promote healthy aging. DR is defined as a reduction in food intake without malnutrition. This nutritional intervention prolongs lifespan in a variety of species [3], while also reducing fertility [4, 5]. DR acts by promoting stress response pathways and preventing age-related functional decline, delaying the appearance of cardiovascular diseases, type 2 diabetes, and neurodegeneration in mammals, including non-human primates [6]. In *C. elegans*, different protocols of DR elicit different degrees of lifespan extension and stress resistance by often distinct and complementary mechanisms [7]. The genetic model of DR – the *eat-2* mutant – has a decreased pumping rate, thus ingesting less bacteria, and requires the FoxA transcription factor PHA-4 [8] and the dimethoxy ubiquinone hydroxylase CLK-1 [9] to extend its lifespan. Distinct and overlapping factors are involved in lifespan extension by bacterial DR (bDR) and liquid DR (lDR) – two protocols of bacterial dilution in liquid cultures. bDR also depends on PHA-4 [8] and is partially dependent on the energy sensing kinase AMPK subunit AAK-2 [7] and on the FoxO transcription factor DAF-16 [8, 10], while lDR depends on the NRF2 transcription factor homolog SKN-1 [11, 12]. The complete absence of bacteria requires the heat shock transcription factor HSF-1 to promote longevity [13], while dilution of medium peptone increases lifespan through AAK-2 and DAF-16 [7], and serial dilution of bacteria on semi-solid medium depends on the same factors in addition to CLK-1 [7, 14]. Stress resistance induced by amino acid restriction in mice [15, 16] and lifespan extension induced by DR or by inhibition of the worm homolog of the nutrient sensing kinase mTOR (LET-363) in *C. elegans* [17] also require the General Control Nonderepressible 2 kinase (GCN2 in mammals or GCN-2 in *C. elegans*).

GCN2 is an evolutionarily conserved serine/threonine kinase that senses amino acid restriction through binding to uncharged tRNAs [18]. In most metazoans, GCN2 is one of the four known kinases that phosphorylate eukaryotic initiation factor 2, α subunit (eIF2α) to inhibit translation initiation [19]. This leads to translation of an alternative and functional open reading frame of the *ATF4* gene (*atf-5* in *C. elegans*) [20, 21]. ATF4 is a transcription factor that targets stress response genes to confer an adaptive cellular response [22]. This pathway is often referred to as the Integrated Stress Response (ISR) pathway, given that it integrates cellular response to multiple stress signals such as nutrient restriction (via GCN2), endoplasmic reticulum stress (via PERK), viral infection (via PKR) and metal deprivation (via HRI) [19, 22, 23]. Thus, GCN2-induced ISR activation in response to amino acid starvation serves as an important interface between nutritional cues and cellular resilience [24].

The mechanism of activation of GCN2 has been well characterized in *S. cerevisiae*, where Gcn2 is the only kinase that phosphorylates eIF2α. For Gcn2 to be activated in vivo it must interact with Gcn1 through its RWD domain, when both proteins are tethered to the ribosome [25]. It is thought that Gcn1 functions to transfer to Gcn2 uncharged tRNAs that enter the A site of the ribosomes, thus activating it [25]. Genetic studies in yeast revealed Yih1 (Yeast IMPACT Homolog 1) as a potent suppressor of Gcn2 function [26]. Yih1 and its mammalian homolog IMPACT harbor a RWD domain that competes with Gcn2/GCN2 for the binding to Gcn1/GCN1 [27, 28]. Yih1 or IMPACT overexpression in yeast or mammalian cells, respectively, abrogates amino acid restriction-induced eIF2α phosphorylation [26, 28]. Like the other members of the GCN2 pathway, Yih1/IMPACT is found in virtually all eukaryotes and in all cells examined [29–31]. In mice, IMPACT is especially abundant in neurons [28, 32]. In these cells, down-regulation of IMPACT inhibits in vitro neuritogenesis by enhancing the basal levels of GCN2 activation, suggesting an important role for IMPACT in neuronal development [32]. It was less clear, however, if IMPACT plays any role in organismal biology. Our hypothesis in the present study was that IMPACT acts to limit stress response at the whole organism level, hence counteracting the beneficial effects of DR and favoring age-related dysfunction. We used *C. elegans* to test this hypothesis and found that the worm homolog of IMPACT (henceforth named IMPT-1) is an inhibitor of GCN-2 that suppresses eIF2α phosphorylation even during fed states. *impt-1* knockdown led to activation of the ISR, improved stress resistance, reduced fertility, decreased food intake, and extended longevity in the worms. These phenotypes resembled DR in all aspects, but DR was additive to *impt-1* RNAi to extend lifespan. Consistent with a DR mimetic, *impt-1* down-regulation activated key downstream players in the DR pathway. Our results highlight IMPT-1 as an important negative regulator of longevity in *C. elegans*, setting up the stage for a better characterization of its functions in mammalian aging and providing a new target for lifespan regulation.

## Results

### Identification and functional characterization of the *C. elegans* IMPACT homolog

To identify the worm homolog of IMPACT, we browsed the *C. elegans* proteome for hits with close similarity to *Mus musculus* IMPACT protein. This analysis led us to *C. elegans* Y52B11A.2, which shared 33 % amino acid identity and 51 % amino acid similarity (E value = 7e^–55^) with mouse IMPACT (Additional file 1: Figure S1a) and displayed the UPF0029 and RWD domains (Additional file 1: Figure S1b), which are conserved among IMPACT proteins across the evolutionary spectrum. We henceforth named this protein IMPT-1 and its gene *impt-1*. A loss-of-function mutation in the *impt-1* gene [33] rendered dead larvae when in homozygosis (data from the *Caenorhabditis* Genetics Center and our own observations). We therefore studied the heterozygous worms – a balanced strain called VC2511 or *impt-1* ^*+/–*^ – and wildtype worms treated with *impt-1* RNAi. *Impt-1* mRNA levels were reduced by 77 % in *impt-1* ^*+/–*^ mutants and by 84 % upon *impt-1* RNAi in comparison to their respective controls (Additional file 2: Figure S2).

**Fig. 1:**
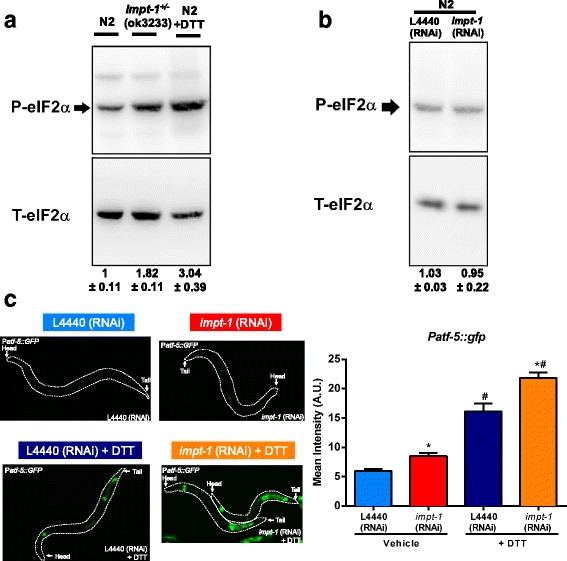
*Impt-1* knockdown activates the integrated stress response (ISR). **a** Immunoblotting of phosphorylated (P-eIF2α) and total eIF2α (T-eIF2α) in pools of day 1 adult N2 and *impt-1* ^*+/–*^ mutant worms. DTT is a PERK activator used as a positive control to induce eIF2α phosphorylation [67]. **b** Same as **a**, but samples are from N2 worms that were maintained in control vector (L4440) or *impt-1* RNAi from eggs. These experiments were repeated twice. Mean ± SEM of quantification is shown below the representative results. **c** Representative images showing the GFP expression of day 0 adult *Patf-5::GFP* reporter strains treated with L4440 or *impt-1* RNAi from L1 larval stage. Worms were exposed for 3 hours with 5 mM DTT or vehicle prior to imaging. *Dashed lines* delimitate the body of the worm. Head and tail of worms are indicated by *arrows*. Average of mean GFP intensity per worm is shown in *right panel* (*n* = 15; *P < 0.0001 to L4440 RNAi; ^#^ *P* < 0.0001 to vehicle treated worms, two-way ANOVA). Data are presented as mean ± SEM. The experiment is representative of two independent trials

Both in yeast and mouse cells, Yih1/IMPACT acts as an inhibitor of Gcn2/GCN2 [26, 28, 32]. We therefore hypothesized that *impt-1* inhibition could activate downstream players in the GCN-2/ISR pathway in *C. elegans*. Consistent with this hypothesis, phosphorylation of eIF2α was 1.82 ± 0.11 fold higher in *impt-1* ^*+/–*^ mutants than in wildtype N2 worms (Fig. 1a), but *impt-1* RNAi did not elicit the same effect (Fig. 1b). However, *impt-1* RNAi increased ATF-5 expression by 40 % (Fig. 1c). Moreover, upregulation of ATF-5 by *impt-1* RNAi was additive to acute incubation with dithiothreitol (DTT) – a PERK activator – suggesting that IMPT-1 may act in parallel with PERK to regulate ATF-5 levels (Fig. 1c). Together, these results demonstrate that Y52B11A.2 is the IMPACT homolog of *C. elegans* and that this protein inhibits the ISR pathway.

### *Impt-1* inhibition extends lifespan and confers stress resistance

**Fig. 2:**
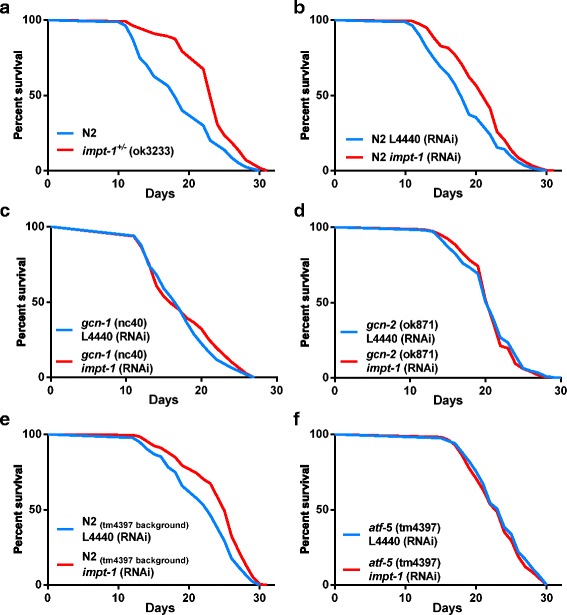
Components of the GCN-2 branch of the integrated stress response (ISR) pathway are required for *impt-1* knockdown-induced longevity. **a** Lifespan assays of N2 and *impt-1* ^*+/–*^ *(ok3233)* mutants. (**b**–**f**) Lifespan assays on control (L4440) or *impt-1* RNAi from L1: (**b**) N2 from CGC, (**c**) *gcn-1(nc40)*, (**d**) *gcn-2(ok871)*, (**e**) N2 from the Blackwell lab [control of *atf-5(tm4397)*], and (**f**) *atf-5(tm4397).* Values of median lifespan and statistics are reported in Additional file 10: Table S1. Survival curves were compared using the log-rank test. All experiments were repeated at least twice. Data demonstrate a representative experiment (experiment I in **a**) or the composite of multiple experiments (II–IV in **b** and **c**, II and IV in **d**, or V–VI in **e** and **f**)

Activation of GCN-2 is required for longevity induced by DR in *C. elegans* and efficient stress response induced by amino acid restriction in mice [16, 17]. Given the observation that *impt-1* knockdown is sufficient to activate the ISR (Fig. 1) and that GCN-2 is required to control basal levels of eIF2α phosphorylation [17], we asked whether *impt-1* abrogation could resemble DR effects on longevity and stress response, and whether these effects were *gcn-2* dependent. *Impt-1* ^*+/–*^ mutants and *impt-1* RNAi-treated worms lived approximately 21 % and 16 % more than their respective controls (Fig. 2a, b, e). Importantly, lifespan extension by *impt-1* RNAi was dependent on multiple components of the GCN-2 branch of the ISR pathway, including *gcn-2*, *gcn-1* and *atf-5* (Fig. 2c, d, f). Whole body reduction of *impt-1* was necessary to increase worm lifespan, since tissue-specific *impt-1* RNAi in the intestine, muscle or neurons did not change lifespan or even reduce it (Additional file 3: Figure S3).

To assess the responsiveness of stress pathways in *impt-1* knockdown models, we measured thermotolerance and oxidative stress resistance. Upon chronic, mild temperature stress (28 °C), *impt-1* ^*+/–*^ mutants lived 22 % longer than wildtype worms, while *impt-1* RNAi-treated worms lived 8 % longer than their controls (Additional file 4: Figure S4a, b). Furthermore, 8-hour exposure to the pro-oxidant agent sodium arsenite at 5 mM or 7.5 mM revealed that *impt-1* ^*+/–*^ mutants were more resistant to oxidative damage than N2 wildtype and *gcn-2* mutant worms (Additional file 4: Figure S4c). This phenotype was also observed in N2 worms treated with *impt-1* RNAi, and it was completely abrogated when *impt-1* was silenced in *gcn-2* mutants (Additional file 4: Figure S4d). Interestingly, *gcn-2* mutants were more resistant to 7.5 mM arsenite than N2 worms (Additional file 4: Figure S4c), demonstrating a baseline stress resistance phenotype as observed in GCN2 knockout mice [16]. Together, these data show that *impt-1* knockdown promotes longevity and stress resistance in a *gcn-2* dependent manner.

**Fig. 3:**
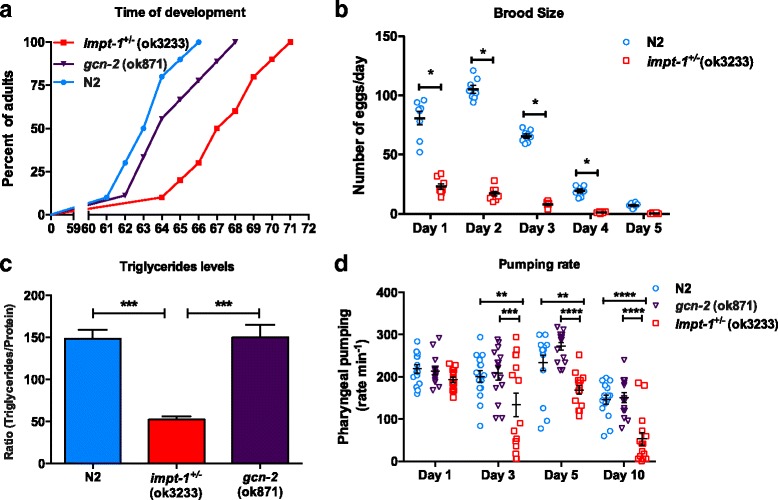
*Impt-1* ^*+/–*^ *(ok3233)* mutants exhibit phenotypes that resemble dietary restriction. **a** Developmental timing after egg laying (*n* = 100 per group; *P* < 0.05; log-rank test). This is representative of two independent experiments. **b** Number of eggs laid during the reproductive period (days 1–5; *n* = 8 per group; *P < 0.05; two-way ANOVA, Sidak post-hoc). Bars are mean ± SEM. This is a representative experiment of two independent experiments. **c** Triglyceride levels normalized by protein levels at day 1 of adulthood (*n* = 3 pools of at least 150 worms per group, ***P < 0.001; one-way ANOVA, Dunnet post-hoc). Data are presented as mean ± SEM. Each pool was obtained in independent experiments. **d** Pharyngeal pumping rate on different days of adulthood (*n* = 15 per group; **P < 0.01,***P < 0.001, ****P < 0.0001; two-way ANOVA, Tukey post-hoc). Data are presented as mean ± SEM. This is a representative experiment of two independent experiments

Other features of DR are reduced food intake, delayed development, reduced fertility, and decreased lipid storage [4, 34–37]. Larval development of *impt-1* ^*+/–*^ mutants was delayed in approximately 4 hours in comparison to N2 worms (Fig. 3a, *P* < 0.0001). The same was observed in *gcn-2* mutants, although with a lesser magnitude (Fig. 3a and Additional file 5: Figure S5a, *P* < 0.0001). In contrast with the *impt-1* ^*+/–*^ mutants, *impt-1* RNAi mildly accelerated development (Additional file 5: Figure S5a). We also scored brood size of *impt-1* ^*+/–*^ mutants and observed an 82 ± 1 % reduction in progeny (Fig. 3b). Again, this phenotype could not be mimicked by *impt-1* RNAi (Additional file 5: Figure S5b). Furthermore, lipid accumulation in *impt-1* ^*+/–*^ mutants was reduced by 65 ± 1 % on day 1 of adulthood compared to N2 (Fig. 3c). Interestingly, this reduction was accompanied by a decrease in pharyngeal pumping observed on days 3, 5, and 10 of adulthood (Fig. 3d), indicating reduced feeding behavior. These parameters were not changed in *gcn-2* mutants (Fig. 3c, d). Although a reduction in food intake may account for the lower lipid levels of *impt-1* ^*+/–*^ mutants, this is not sufficient to explain it since N2 worms treated with *impt-1* RNAi also exhibited decreased pharyngeal pumping but no differences in lipid levels on day 1 of adulthood (Additional file 5: Figure S5c, d). In summary, *impt-1* knockdown resembles many features of DR such as extended longevity, increased stress resistance, and reduced food intake. Delayed development, reduced brood size, and decreased fat accumulation is also observed when one allele of *impt-1* is knocked out, but not when *impt-1* is downregulated using RNAi.

### *Impt-1* limits lifespan extension conferred by DR

**Fig. 4:**
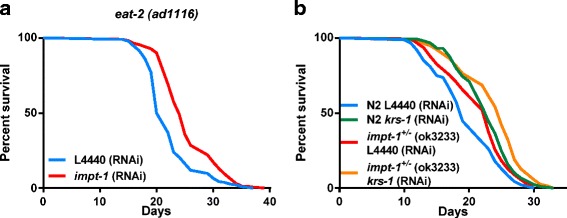
Longevity of *impt-1* knockdown models is additive to dietary and amino acid restriction*.* **a** Lifespan assays of *eat-2(ad1116)* worms treated with L4440 (control) or *impt-1* RNAi from L1. **b** Lifespan assays of N2 and *impt-1* ^*+/–*^ worms treated with L4440 or *krs-1* RNAi from day 0 of adulthood. Values of median lifespan and statistics are reported in Additional file 10: Table S1. Survival curves were compared using the log-rank test. All experiments were repeated twice. Data demonstrate the composite of experiments VII–VIII (**a**) or IX–X (**b**)

Next, we investigated whether DR and *impt-1* knockdown had an additive effect on longevity. When maintained in *impt-1* RNAi, *eat-2* worms lived 25 % more than worms maintained in the control RNAi (Fig. 4a). Moreover, silencing of lysil-tRNA synthetase (*krs-1*) using RNAi – a model of amino acid restriction [17] – increased the lifespan of N2 worms to an extent that closely resembles the longevity of *impt-1* ^*+/–*^ mutants, while further increasing the lifespan of these mutants (Fig. 4b). Moreover, longevity induced by *krs-1* RNAi was blocked in *gcn-1* mutants (Additional file 6: Figure S6). Considering that DR [17] and *impt-1* knockdown need a functional GCN-2 pathway to produce their effects, these results suggest that IMPT-1 is a rate-limiting protein for DR-induced lifespan extension by inhibiting GCN-2.

### Components of the DR pathway are activated by *impt-1* knockdown in an age-dependent manner

**Fig. 5:**
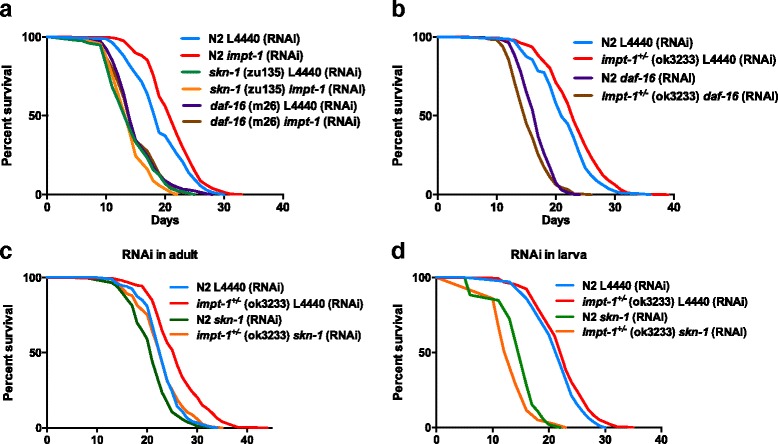
*Impt-1* knockdown interacts in an age-dependent manner with multiple components of the DR pathway to control longevity*.* **a** Lifespan assays of N2 worms, *skn-1(zu135)*, and *daf-16(m26)* mutants treated with L4440 (control) or *impt-1* RNAi from L1. **b** Lifespan assays of N2 worms and *impt-1* ^*+/–*^ *(ok3233)* mutants treated with L4440 or *daf-16* RNAi from day 0 of adulthood. **c**, **d** Lifespan assays of N2 worms and *impt-1* ^*+/–*^ *(ok3233)* exposed to L4440 (control) RNAi or *skn-1* RNAi from day 0 of adulthood (**c**) or during larval stages (L1–L4) (**d**). Values of median lifespan and statistics are reported in Additional file 10: Table S1. Survival curves were compared using the log-rank test. All experiments were repeated at least three times. Data demonstrate the composite of experiments XI–XIII (**a**), XIV–XVII (**b**), XVIII–XIX (**c**) or XX–XXII (**d**)

As *impt-1* down-regulation mimics features of DR, we investigated whether downstream components of the DR pathway in *C. elegans* were involved in longevity induced by *impt-1* knockdown. First, we tested if *impt-1* RNAi extended lifespan in worms carrying loss-of-function mutations in *aak-2*, *daf-16*, *skn-1*, and *hsf-1*. Surprisingly, all of these genes were necessary for lifespan extension induced by *impt-1* RNAi (Fig. 5a and Additional file 7: Figure S7a,b).

Given that most of these mutations (including in the *impt-1* gene) affect development, this could be the stage in which *impt-1* and these DR genes interact to influence lifespan. Furthermore, developmentally impaired mutants might indirectly mitigate the ability of *impt-1* knockdown to prolong lifespan if this phenotype is simply a manifestation of developmental traits. We therefore tested if silencing these genes and other DR-modulated genes only during adulthood could block longevity induced by *impt-1* knockdown. When DR genes were silenced during the adult life of *impt-1* ^*+/–*^ mutants, we found that *aak-2* RNAi blunted *impt-1* ^*+/–*^ extended longevity, while *let-363* RNAi did not further increase it (Additional file 7: Figure S7c,d). Furthermore, *impt-1* ^*+/–*^ mutants treated with *daf-16* RNAi during adulthood not only failed to exhibit increased lifespan, but lived 7 % less than wildtype worms exposed to the same RNAi (Fig. 5b). However, loss of other DR genes (e.g., *skn-1*, *hsf-1*, and *atf-5*) after development did not affect *impt-1* ^*+/–*^ longevity (Fig. 5c and Additional file 7: Figure S7e, f). The fact that all of the DR-related gene mutations studied here blocked the effects of *impt-1* RNAi treatment starting at L1, but only some DR-related genes affected *impt-1* ^*+/–*^ longevity when silenced during adulthood, suggested to us that *impt-1* might control lifespan by interacting with some genes during adult life (e.g., *daf-16*, *aak-2*, and *let-363*) and others during the larval stages (e.g., *skn-1*, *hsf-1*, and *atf-5*). Consistent with this notion, when N2 and *impt-1* ^*+/–*^ worms were maintained in control or *skn-1* RNAi from L1 to L4 larval stages, and then transferred to plates with control RNAi for the rest of their adult life, larva-restricted *skn-1* RNAi not only blocked lifespan extension of *impt-1* ^*+/–*^ mutants but also reduced it by 12 % in comparison to N2 worms on the same regimen (Fig. 5d).

**Fig. 6:**
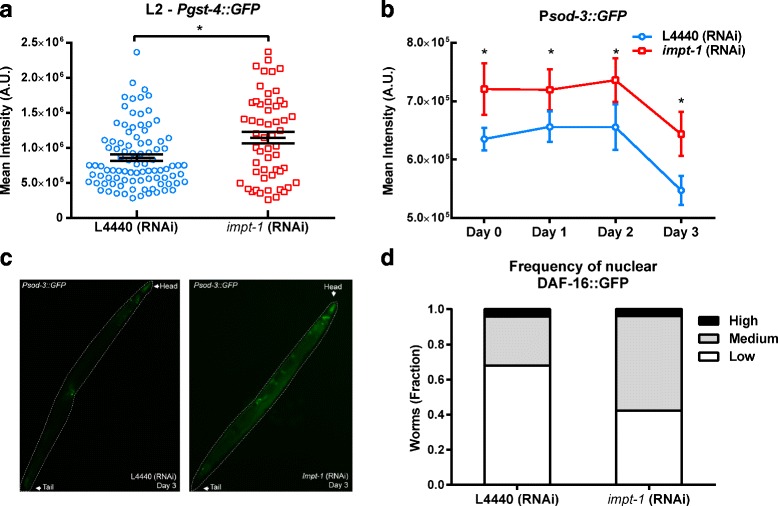
*Impt-1* knockdown activates SKN-1 and DAF-16 at different ages*.* **a** Average mean GFP intensity at L2 stage of the *Pgst-4::GFP* reporter strain treated with L4440 (control) or *impt-1* RNAi since L1 (L4440, *n* = 92; *impt-1*, *n* = 54; *P < 0.001, unpaired Student *t* test). Bars are mean ± SEM. This experiment was repeated three times. **b** Average mean GFP intensity at different days of adulthood of the *Psod-3::GFP* reporter strain treated with L4440 (control) or *impt-1* RNAi from L1 (L4440, *n* = 24–30; *impt-1*, *n* = 22–28 varying depending on the time point; *P = 0.0142 for the RNAi effect, two-way ANOVA). This is a composite of two independent experiments. **c** Representative images of (**b**) at day 3. *Dashed lines* delimitate the body of the worm; head and tail are indicated by *arrows*. **d** Qualitative analysis of the fraction of worms with high, medium, or low levels of nuclear DAF-16::GFP upon incubation with L4440 (control) or *impt-1* RNAi from L1 (L4440, *n* = 25; *impt-1*, *n* = 26; χ^2^ test). Data are presented as mean ± SEM in (**a**, **b**) or as stacked fractions in (**d**)

We therefore investigated whether *impt-1* RNAi could affect SKN-1 function during larval stages. We assessed the expression of a reporter construct in which a SKN-1 target promoter directs the expression of GFP (i.e., P*gst-4::GFP*) [38, 39]. In our first analysis with the P*gst-4::GFP* reporter strain we observed no significant differences in GFP expression in response to *impt-1* silencing, except for a trend towards an increase during L2 (*P* = 0.09, Additional file 8: Figure S8a) that turned out to be significant when the N was augmented (Fig. 6a; representative images are shown in Additional file 8: Figure S8b).

Next, we tested if *impt-1* knockdown affected DAF-16 activity by measuring the expression of P*daf-16::daf-16::GFP* and the DAF-16 target construct P*sod-3::GFP* in response to *impt-1* RNAi. The expression of P*sod-3::GFP* during the first days of adulthood was higher in worms treated with *impt-1* RNAi when compared to control RNAi, with the biggest difference being at day 3 (Fig. 6b, c). The expression of P*sod-3::GFP* in larvae subjected to the same protocol was not different until the final larval stage (L4), demonstrating that DAF-16 activation by *impt-1* knockdown starts at late larval/early adult stages (Additional file 8: Figure S8c). We also observed a trend towards nuclear localization of DAF-16 in day 3 worms under *impt-1* RNAi (Fig. 6d). Taken together, these data demonstrate that several mediators of DR and the activation of stress response transcription factors are necessary to explain longevity induced by *impt-1* knockdown, and this occurs in an age-dependent manner.

Finally, to assess if *impt-1* expression was controlled by these DR-related transcription factors, we measured *impt-1* mRNA in *daf-16*, *skn-1*, and *hsf-1* loss-of-function mutants (Additional file 9: Figure S9a) and in a condition where they were activated, i.e., during DR (Additional file 9: Figure S9b). There were no significant differences whatsoever, indicating that *impt-1* is constitutively expressed under these conditions and is not transcriptionally regulated by these stress-associated transcription factors. Nonetheless, *impt-1* expression decreased with aging regardless of the diet (Additional file 9: Figure S9b) – a feature that could help activation of the ISR in aged individuals.

## Discussion

**Fig. 7:**
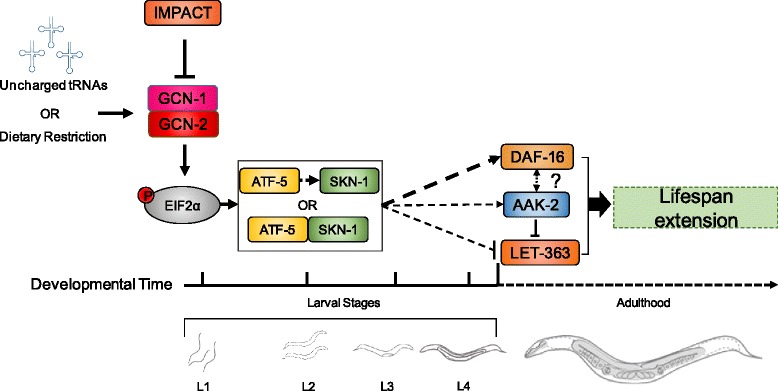
Working model of lifespan extension induced by *impt-1* knockdown in *C. elegans*. IMPACT acts as an inhibitor of GCN-2. *Impt-1* knockdown activates the GCN-2 branch of the ISR, increases the phosphorylation of eIF2α and induces ATF-5 expression. This pathway is further stimulated by an increase in uncharged tRNAs exerted by dietary restriction. In early larval stages, ATF-5 induces SKN-1 or acts in concert with it to activate a stress response program that leads to DAF-16 induction in adults and confers lifespan extension. *Impt-1* also interacts with *hsf-1* during larval stages and with *aak-2* and *let-363* during adulthood to control lifespan

IMPACT is an ancient protein that exerts fundamental roles in the cellular adaptive response to nutritional stress [26, 28, 30, 32]. IMPACT is conserved across the metazoan phylum alongside its interacting partners GCN1and GCN2, through which it negatively influences eIF2α phosphorylation and limits ISR activation in response to amino acid restriction and other stress conditions [28, 30]. Despite its important functions, no previous work had addressed the role of IMPACT in multicellular organisms. Here, we identified the IMPACT homolog of *C. elegans* (IMPT-1) and demonstrated that its partial loss-of-function is sufficient to induce the ISR under the fed state. In turn, *impt-1* knockdown extends lifespan and ameliorates stress response in GCN-2 and ISR dependent manners. These effects also depend on several genes required for lifespan extension promoted by DR in *C. elegans* such as *skn-1*, *daf-16*, and others. These genetic interactions occur in a timely fashion, in which knocking down *skn-1* during larval stages is sufficient to block lifespan extension exerted by *impt-1* knockdown. *Impt-1* silencing induces SKN-1 function in L2 worms, and affects DAF-16 function later in adulthood. These results highlight the pleiotropic functions of IMPT-1 on longevity, where its knockdown during development leads to a chain of events first manifested by ISR up-regulation, followed by SKN-1 activation and later by DAF-16 induction (Fig. 7).

A similar phenomenon is observed when worms are offered excess NAD^+^ starting at late embryonic development [40, 41]. These protocols prolong lifespan by a mechanism that requires activation of the mitochondrial unfolded protein response (UPR^mt^) first observed in day 1 adults and that consequentially leads to DAF-16 translocation and *sod-3* transcription in day 3 adults. Lifespan extension induced by NAD^+^ donors also requires the sirtuin SIR-2.1 and SKN-1 [41, 42], similarly to *impt-1* knockdown-induced longevity (Fig. 5 and data not shown). Interestingly, mitochondrial stress induced by inhibition of certain components of the electron transport chain (i.e., *isp-1* and *clk-1*) promotes eIF2α phosphorylation during development, activates UPR^mt^ and prolongs lifespan in *C. elegans*, and this requires GCN-2 [43]. Moreover, mild mitochondrial stress [44] or stimulation of UPR^mt^ [42] during the larval stages are sufficient to render worms long lived. We thus suggest that multiple metabolic pathways can converge into GCN-2 and the ISR pathway during development to regulate lifespan and IMPT-1, as an inhibitor of the GCN-2 pathway, limits their effects.

How exactly the activation of GCN-2 and the ISR pathway initiates downstream events during development that culminate in lifespan extension is not absolutely clear. Since expressions of the ISR transcription factor ATF-5 and the SKN-1 target gene *gst-4* are more prematurely up-regulated by *impt-1* RNAi and both ATF-5 and SKN-1 are required during larval stages for the longevity phenotype of *impt-1* knockdown models, we propose that these two transcription factors are acting in concert to trigger a longevity pathway. Indeed, in human cells, ATF4 (the mammalian ATF-5 homolog) has been shown to dimerize with NRF2 (the mammalian SKN-1 homolog) to activate the enhancer of the heme oxygenase-1 gene [45]. In *C. elegans*, SKN-1 and ATF-5 are mutually regulated in response to endoplasmic reticulum (ER) or oxidative stress [46]. Importantly, *atf-5* is a SKN-1 target gene and *atf-5* ablation prevents ER stress from inducing transcription of *skn-1* and its target genes [46]. Thus, these studies and our observations provide evidence to suggest that SKN-1 function is controlled by the ISR. However, NRF2 is also a direct substrate of the ER stress-regulated eIF2α kinase PERK, and it has been proposed to mediate PERK-dependent cell survival independently of eIF2α phosphorylation in mouse cells [47]. Whether SKN-1 and ATF-5 act together or in parallel to control lifespan of *C. elegans* is a matter for future studies.

The importance of IMPT-1 and GCN-2 during larval stages is further supported by delayed development in the *impt-1(ok3233)* heterozygous mutants and mid-larval arrest in *impt-1(ok3233)* homozygous mutants and in N2 worms treated with *krs-1* RNAi from eggs ([17] and confirmed by our own data). Given that both *krs-1* and *impt-1* ablations activate ISR when initiated in early development (Fig. 1 and [17]), these interventions could represent a stress signal that inhibits progression through the larval stages [48–50]. However, GCN-2 depletion by RNAi does not reverse the larval arrest phenotype of *impt-1(ok3233)* homozygous mutants (data not shown), indicating independence of GCN-2. Similarly, the knockdown of IMPACT in mouse neuronal cell lines partially affects neuritogenesis in a GCN2-independent manner [32]. In addition, it has been shown that Yih1 controls yeast cell cycle independently of Gcn2 or Gcn1 [51]. These phenotypes are in sharp contrast with the effects of *impt-1* knockdown on longevity and stress resistance, which require both *gcn-2* and *gcn-1*, evidencing the broad, yet poorly understood roles of IMPACT.

*Impt-1* knockdown by RNAi or by its heterozygous null mutation render worms with very similar phenotypes but also with some fundamentally different ones. Both interventions decrease *impt-1*, reduce pharyngeal pumping, increase lifespan, and promote stress resistance at similar levels. On the other hand, while *impt-1* ^*+/–*^reduces brood size, delays development and decreases triglyceride content, *impt-1* RNAi does not affect these parameters, or in the case of developmental timing even accelerates it. *Impt-1* RNAi also does not promote eIF2α phosphorylation as *impt-1* ^*+/–*^ does, at least not in the time point that we analyzed, but it increases ATF-5 expression, which is directly downstream of eIF2α. These distinctions may be explained by different hypotheses, including maternally inherited traits, RNAi delivery constraints or timing. More importantly, they highlight the fact that these phenotypes are not completely associated, and that *impt-1* knockdown can increase lifespan without continuous hyperphosphorylation of eIF2α or affecting fertility, development, and fat accumulation.

In a broad spectrum, the phenotypes of *impt-1* knockdown models can resemble the characteristics of dietary restricted animals, i.e., increased lifespan, improved stress response, delayed development, reduced fertility, decreased fat accumulation, and diminished food intake. Additionally, the levels of IMPT-1 and uncharged tRNAs seem to limit each other’s effects. For example, when *impt-1* is decreased (e.g., in *impt-1* ^*+/–*^ mutants or *impt-1* RNAi) but uncharged tRNAs levels are low (e.g., during the fed state), lifespan extension is limited, as observed when uncharged tRNA levels increase (e.g., during DR) in the presence of high levels of IMPT-1 (e.g., in N2 worms or control RNAi). However, when DR and *impt-1* knockdown are combined, lifespan is extended to its maximum under these circumstances. This could be interpreted through the perspective that DR and *impt-1* knockdown act in parallel pathways. However, the genetic interaction of *impt-1* with essentially all downstream components of the DR pathway suggests otherwise, that DR and *impt-1* indeed share the same route to control lifespan. One alternative explanation is that *impt-1* knockdown releases more GCN-1 to interact with GCN-2 and respond more efficiently to uncharged tRNAs that are more abundant during DR. Therefore, loss of *impt-1* would have an effect under the fed state, and this effect would be exacerbated under DR. Considering that *impt-1* knockdown (Fig. 2), *eat-2* [17] and *krs-1* RNAi (Additional file 6: Figure S6) all increase lifespan in GCN-2 or GCN-1 dependent manners, we suggest that these interventions converge into activating GCN-2 to exert their effects.

However, how can *impt-1* knockdown increase GCN-2 activation under the fed state? A similar effect was observed in mouse neuronal cells, which express high levels of IMPACT. The knockdown of IMPACT in these cells resulted in increased levels of phosphorylated GCN2, the active form of the kinase, and of phosphorylated eIF2α [32]. It is possible that the resulting increase in GCN-1 available for interacting with GCN-2 will render GCN-2 more sensitive to the basal levels of uncharged tRNAs that occur naturally in the cells, as a result of each cycle of translation elongation as they exit the ribosomes.

And why would DR and *krs-1* RNAi increase lifespan when applied to adult worms while *impt-1* knockdown requires activation of SKN-1 during development? One possible explanation is a model where stoichiometric balance determines the effects. In such a model, uncharged tRNA levels are sufficiently high during development (perhaps due to increased protein synthesis) and IMPT-1 becomes rate limiting, exerting an important inhibitory pressure on GCN-2. On the other hand, uncharged tRNAs are less abundant in adults and therefore IMPT-1 is dispensable for GCN-2 regulation in the absence of a proper stimulus. This model is supported by the important functions of IMPT-1 during development and by the fact that *krs-1* RNAi during adulthood adds to *impt-1* ^*+/–*^ to prolong lifespan. Curiously, while *krs-1* RNAi applied to adult N2 worms increases lifespan in our hands, another study reported decreased lifespan of N2 worms under the same RNAi clone [17]. These discrepancies might be related to slightly different protocols that could sensitize or desensitize the GCN-2 pathway. One such difference in our study is the use of FUdR, which inhibits progeny.

Reduced food intake is usually the primary cause of DR. The fact that *impt-1* knockdown causes the pharyngeal pumping rate to be decreased indicates that this phenotype may also explain or at least partially contribute to longevity. Interestingly, brain-specific activation of GCN2 and phosphorylation of eIF2α leads to an aversive behavior against amino acid deficient diets [52–54]. Hyperactivation of the GCN2 pathway may therefore represent an evolutionarily conserved hub to control food intake and metabolic adaptation in response to changes in nutrient availability.

## Conclusions

In summary, reduction of IMPT-1 in worms is sufficient to trigger the ISR pathway and confer phenotypic characteristics that resemble DR, including extended longevity. Further studies are required to confirm the importance of IMPT-1 in vertebrates, but its highly conserved function and the broad evolutionary spectrum where the IMPACT/GCN2 pathway actuates gives us hints that our findings might be applied to more complex organisms. If so, IMPACT may serve as an important drug target to mimic DR in humans, in a way that could dissociate its beneficial effects from its heavy demands.

## Methods

### Identification of IMPACT in *C. elegans*

The *C. elegans* homolog of mouse IMPACT was identified using Blastp from NCBI. We browsed proteins in the *C. elegans* genome (Taxid: 6239) sharing sequence-similarity with *Mus musculus* IMPACT (GenBank: BAA35139.1) and analyzed predicted domains of Y52B11A.2 gene product of *C. elegans* by DELTA-BLAST from NCBI.

### Construction of the *impt-1* RNAi vector

Construction of *impt-1* RNAi plasmid was similar to that reported elsewhere [55, 56]. A region corresponding to an exonic part of the *impt-1* gene of N2 worms was amplified by PCR using the forward primer 5′-AA**TCTAGA**CTGCCGGGCAACACATAAT-3′ and the reverse primer 5′-AA**CTCGAG**TATCCGCTTTCATTCGATCC-3′. The resulting PCR product was cloned into the L4440 vector using the XbaI and XhoI sites (regions in bold) and the Quick Ligation kit (New England Biolabs). Plasmids were transformed into *E. coli* TOP10 (One Shot® iTOP10 Chemically Competent *E. coli*). Recombinant clones were selected using ampicillin. Plasmids were extracted using QIAGEN Plasmid Mini Kit and sequenced to confirm the presence of the insert. Plasmids were used to transform *E. coli* HT115(DE3), which was used to feed the nematodes.

### Strains and maintenance of *C. elegans*

*C. elegans* strains were cultured at 20 °C in streptomycin-supplemented (100 μg/mL) Nematode Growth Medium (NGM) with a lawn of *E. coli* OP50-1 as the food source, unless stated otherwise [57]. The following strains were obtained from the *Caenorhabditis Genetics Center* (CGC): wild-type N2 Bristol, DA1116 *eat-2(ad1116)*, RB976 *gcn-2(ok871)*, ST60 *gcn-1(nc40)*, VC2511 *impt-1(ok3233) I/hT2 [bli-4(e937) let-?(q782) qIs48]*, MR507 *aak-2(rr48)*, DR26 *daf-16(m26)*, EU31 *skn-1(zu135)*, PS3551 *hsf-1(sy441)*, VP303 *rde-1(ne219);kbIs7[nhx-2p::rde-1 + rol-6(su1006)]*, NR350 *rde-1(ne219);kzIs20[(hlh-1p::rde-1) + pTG95(sur-5p::NLS::GFP)]*, TU3401 *sid-1(pk3321);uIs69[pCFJ90(myo-2p::mCherry) + unc-119p::sid-1]*, CF1553 *muIs84[(pAD76) sod-3::GFP + rol-6]*, TJ356 *zIs356 [daf-16p::daf-16a/b::GFP + rol-6]*, and CL2166 *dvIs19[(pAF15)gst-4p::GFP::NLS]*. The strains *atf-5(tm4397)*, its respective N2 control, and the *Patf-5::GFP* transgenic line were kindly provided by Dr. Keith Blackwell and Dr. David Ron.

#### RNAi

For RNAi experiments, NGM was supplemented with ampicillin (100 μg/mL), tetracycline (12.5 μg/mL), and isopropyl β-D-1-thiogalactopyranoside (IPTG; 1 mM) and seeded with HT115(DE3) *E. coli* (OD 1.5 concentrated 10×). Expression of dsRNA was induced by addition of 1 mM IPTG to concentrated bacteria right before seeding the plates [58].

#### Western blotting

*C. elegans* protein lysates were obtained using approximately 700 synchronized day 0 adults. For positive control, drops of 5 mM DTT or vehicle were applied onto the worms and incubated at 20 °C for 3 hours. Worms were washed with sterile Milli-Q water and after decantation, the supernatant containing water and bacteria was removed. RIPA buffer (50 mM Tris pH:8.0, 150 mM NaCl, 1 % NP-40, 0.5 % sodium deoxycholate, 0.1 % SDS) was added and worms were shattered using metallic beads (Bullet Blender, Next Advance). Beads were removed and samples centrifuged at 17950 × *g* for 10 min at 4 °C. Supernatant was removed to obtain protein-containing lysates. Protein quantitation was performed using the BCA kit (Thermo Fisher). Immunodetection of phosphorylated eIF2α and total eIF2α was performed as described previously [51]. The antibodies anti-Ser(P)52-eIF2α (Invitrogen, 44728G) and anti-Sui2 (eIF2α) [59] were used to detect phosphorylated and total eIF2α, respectively.

#### Lifespan assays

Approximately 150 synchronized young-adult worms were transferred to lifespan plates (NGM supplemented with 50 μM FUdR) containing OP50-1 or HT115(DE3) bacteria. For RNAi, worms were kept in control RNAi (L4440) or *impt-1* RNAi starting from L1, and then transferred as young adults to lifespan plates containing the same bacteria as the initial food source. For RNAi only in adults, worms were kept in OP50-1 and then transferred as young adults to lifespan plates with HT115(DE3) expressing control vector or *impt-1* RNAi. For RNAi in larvae, worms were kept in control RNAi or *skn-1* RNAi and then transferred as young adults to lifespan plates with HT115 expressing control RNAi. Plates were maintained at 20 °C and monitored daily [60]. For heat stress experiments, adult worms were kept at 28 °C. Dead worms were scored until the death of the last worm and the ones that crawled out of the agar, or showed bag-of-worms or exploded vulva were censored.

#### Brood size analysis

Brood size experiments were similar to previous reports [61]. Synchronized hermaphrodites were maintained in OP50-1 or HT115(DE) expressing control vector or *impt-1* RNAi and then transferred individually at the L4 stage to a well of a 24-well plate containing NGM media and the same bacteria used during development. Worms were transferred daily to a new well and scored until the end of the reproductive phase. After 24 h, for each day, larvae and eventually dead eggs that were laid by each worm were counted. Eight worms were used in each replicate.

#### Triglyceride quantification

Plates containing hundreds of synchronized day 1 adult worms were washed with 2 mL of sterile milli-Q water and transferred to Eppendorf conical tubes. After decantation, the supernatant containing water and bacteria was removed and 250 μL of a 5 % NP-40 solution was added. The worm suspension was heated at 75 °C for 10 minutes in a thermoshaker at 800 rpm. Samples were then vortexed and cooled at room temperature for 10 minutes. To eliminate the insoluble particles, tubes were centrifuged at 12000 × *g* for 5 minutes at 4 °C. The supernatant containing the lipids was then transferred to a new tube for triglyceride quantification. Triglyceride quantitation was performed using the LabTest kit according to the manufacturer’s instructions. Lipid extract (2 μL) or standard control (200 mg/dL) were added to 200 μL of detection buffer and the solution was heated at 37 °C for 10 minutes [62]. The product was quantified at 490 nm using a plate reader (EL808 Ultra Microplate Reader). Concentration of triglycerides was estimated according to the standard control and then normalized by protein levels, as measured by the BCA kit using the same extract.

#### Developmental timing assay

Time of development was performed as previously described [63], with modifications. Briefly, several adult hermaphrodites were transferred to NGM plates containing OP50-1 or HT115 expressing *impt-1* RNAi or control vector and let to lay eggs for 1–12 hours, depending on the experiment. Adults were then killed and, in some cases, when there were too many eggs on the plate, the eggs were transferred to new plates. Plates were monitored every 6 hours until the appearance of the first adult of the progeny and then every hour. Adults were counted and removed until all the worms reached adulthood.

#### Pumping analysis

Synchronized worms were maintained in NGM containing OP50-1 or HT115(DE3) for RNAi experiments. Pharyngeal pumping was scored at different developmental stages, as indicated. Pharynx movements were observed using a light stereoscope, and counted during a period of 10 seconds. Values were multiplied by 6 to the obtain pumping min^–1^ rate. At least two replicates of each developmental stage were performed and 10 worms were scored per day [64].

#### GFP reporter analysis

Synchronized L1 reporter strains (P*atf-5::GFP*, P*sod-3::GFP*, P*gst-4::GFP* or P*daf-16::daf-16::GFP*) were maintained in control or *impt-1* RNAi from L1 stage [40, 65]. Worms were transferred to a well of a 96-well plate containing 50 μL of M9 media (22 mM Na_2_HPO_4_, 22 mM KH_2_PO_4_, 85 mM NaCl, 1 mM MgSO_4_) at different time points, and immobilized with 0.1 % sodium azide right before images were acquired using the InCell Analyzer 2200 microscope (GE HealthCare). For *atf-5* expression, drops of 5 mM DTT or vehicle were applied onto young adult worms for 3 hours prior to imaging. Fifteen worms of each group were transferred to new plates and photographed using a SteREO DiscoveryV8 modular stereoscope (Zeiss). Images were analyzed using ImageJ and mean intensity or integrated density of each worm was calculated. For the P*daf-16::daf-16::GFP* construct, the frequency of worms with high; medium or low levels of nucleated DAF-16::GFP were assessed qualitatively by a blind investigator. The worms were classified based on the intensity of nuclear DAF-16::GFP and the number of cells with nucleated versus cytoplasmic DAF-16::GFP.

#### RT-qPCR

Approximately 150 synchronized day 0 worms were collected and the total RNA was extracted using the TRIzol Reagent (Life Technologies), following the manufacturer’s instructions. RT-qPCR was performed as previously described [66]. Briefly, the synthesis of cDNA was conducted using the High-Capacity cDNA Reverse Transcription kit (Life Technologies) using 1 μg of total RNA. After cDNA synthesis, gene expression was quantified by real-time PCR in an ABI 7000 detection system (Applied Biosystems) using the Maxima SYBR-Green Master Mix (Fermentas). The following primers were used: *impt-1* forward 5′-CGAGGTTCGGAGTAAGGAAG-3′, *impt-1* reverse 5′-CCCCATCATCCACACAATC-3′, *his-10* foward 5′-GCAATTCGTCGTCTCGC-3′ and *his-10* reverse 5′-GACTCCACGGGTTTCCT-3′. *Impt-1* expression was normalized to *his-10* expression. Specificity of amplification was tested by melting analysis.

#### Statistics

For lifespan and heat stress analyses, survival comparisons between groups were made using the log-rank test. For comparisons between two means, a two-tailed Student *t* test was performed. Variance was tested and a non-parametric test was used when variance was different between groups. For more than two means, one-way analysis of variance (ANOVA) was conducted. For experiments with two independent variables, means were compared by two-way ANOVA. When no statistic interaction was observed, an overall independent variable effect was reported. In cases where statistic interaction between variables was detected, multiple comparison post-tests were performed and main effects reported. Some means were composed by pooled data from all replicates and are indicated as so. Comparisons were made using Graph Pad Prism 6 and null hypothesis was rejected when *P* < 0.05.

## Additional files

Additional file 1: Figure S1. The IMPACT protein of *Caenorhabditis elegans*. (a) Alignment between the amino acid sequences of mouse protein IMPACT (sbjct) and Y52B11A.2 of *C. elegans* (query). Result show 33 % identity and 51 % similarity between both proteins (E value of 7e^–55^). (b) Predicted domains of *C. elegans* Y52B11A.2. (PDF 270 kb)
Additional file 2: Figure S2.
*Impt-1* expression in *impt-1* ^*+/–*^ mutants and worms exposed to *impt-1* RNAi. (a,b) *Impt-1* mRNA expression in day 0 N2 (*n* = 3 pools of 150 worms) and *impt-1* ^*+/–*^ *(ok3233)* (*n* = 3 pools of 150 worms) worms (a) or N2 worms treated with L4440 (control) (*n* = 3 pools of 150 worms) or *impt-1* RNAi (*n* = 3 pools of 150 worms) from eggs (b). Data are presented as mean ± SEM and compared using unpaired Student *t* test, ***P* = 0.0097 in comparison to N2, **P* = 0.036 in comparison to L4440. The experiments were performed once in triplicate. Raw data in Additional file 11: Table S2. (PDF 296 kb)
Additional file 3: Figure S3. Tissue-specific *impt-1* RNAi in intestine, muscle, or neurons is not sufficient to increase lifespan. (a–c) Lifespan assays of worms with tissue-specific RNAi sensitivity in intestine (a), muscle (b), or neurons (c). The worms were treated with L4440 (control) or *impt-1* RNAi from L1. Values of median lifespan and statistics are reported in Additional file 10: Table S1. Survival curves were compared using the long-rank test. All experiments were repeated three times. Data demonstrate the composite of experiments XXIII–XXV (a), XXVI–XXVIII (b), or XXIX–XXXI (c). (PDF 52 kb)
Additional file 4: Figure S4.
*Impt-1* knockdown promotes stress resistance. (a, b) Lifespan assays under heat stress (28 °C) of N2 worms and *impt-1* ^*+/–*^ *(ok3233)* mutants (a) or N2 worms treated with L4440 (control) or *impt-1* RNAi from L1 (b). Values of median lifespan and statistics are reported in Additional file 10: Table S1. Survival curves were compared by log-rank test. Experiment (a) was repeated three times and (b) was performed once. Data demonstrate the composite of experiments XXXII–XXXIV (a) or the experiment XXXV (b). (c) Fraction of dead N2, *impt-1* ^*+/–*^ *(ok3233)* and *gcn-2(ok871)* worms when exposed to 5 mM or 7.5 mM sodium arsenite for 7 hours (N2, *n* = 44; *impt-1* ^*+/–*^, *n* = 46; *gcn-2*, *n* = 48; **P* < 0.05; One-way ANOVA, Tukey post-hoc). This is a representative experiment of two independent experiments. (d) Fraction of dead worms after treatment with 5 mM or 7.5 mM sodium arsenite for 7 hours. This is a representative experiment of two independent experiments (N2 L4440 RNAi, *n* = 47; N2 *impt-1* RNAi, *n* = 46; *gcn-2* L4440 RNAi, *n* = 46; *gcn-2 impt-1* RNAi, *n* = 44; **P* < 0.05; Two-way ANOVA, Tukey post-hoc). RNAi was initiated at L1. Data are presented as mean ± SEM. (PDF 303 kb)
Additional file 5: Figure S5. Phenotypic characterization of N2 worms treated with *impt-1* RNAi from L1*.* (a) Developmental timing after egg laying of N2 and *gcn-2(ok871)* worms (N2 L4440 RNAi, *n* = 469; N2 *impt-1* RNAi, *n* = 510; *gcn-2* L4440 RNAi, *n* = 365; *gcn-2 impt-1* RNAi, *n* = 411; *P* < 0.05; log-rank test; all groups are statistically different compared to each other). This is the composite of three experiments*.* (b) Brood size (N2 L4440 RNAi, *n* = 24; N2 *impt-1* RNAi, *n* = 24 **P* < 0.05, Two-way ANOVA, Bonferroni post-hoc). This is a composite of three independent experiments. (c) Pharyngeal pumping rate at different ages [L4 (L4440, *n* = 30; *impt-1*, *n* = 30), Day 0 (L4440, *n* = 30; *impt-1*, *n* = 30), Day 1 (L4440, *n* = 50; *impt-1*, *n* = 50), Day 3 (L4440, *n* = 19; *impt-1*, *n* = 26); **P* < 0.05, Two-way ANOVA, Sidak post-hoc)]. Each time point was scored at least twice and the data is the composite of five experiments. (d) Triglyceride levels at day 1 of adulthood normalized by protein levels (*n* = 4 pools of at least 150 worms; unpaired Student *t* test). Bars are presented as mean ± SEM. Each pool was obtained in independent experiments. (PDF 274 kb)
Additional file 6: Figure S6. Lifespan increase induced by *krs-1* RNAi is blocked in *gcn-1* mutants*.* Lifespan assay of N2 worms and *gcn-1(nc40)* mutants treated with L4440 or *krs-1* RNAi from day 0 adults. Values of median lifespan and statistics are reported in Additional file 10: Table S1. Survival curves were compared using the log-rank test. Data demonstrate the composite of experiments XXXVI and XXXVII. (PDF 146 kb)
Additional file 7: Figure S7.
*Impt-1* interactions with components of the DR pathway to control longevity. (a,b) Lifespan assays of *aak-2(n48)* and *hsf-1(sy441)* mutants treated with L4440 (control) or *impt-1* RNAi from L1. (c–f) Lifespan assays of N2 worms and *impt-1* ^*+/–*^ *(ok3233)* mutants treated with *aak-2*, *let-363*, *hsf-1*, or *atf-5* RNAi from day 0 adults. Values of median lifespan and statistics are reported in Additional file 10: Table S1. Survival curves were compared using the log-rank test. All experiments were repeated at least twice. Data demonstrate the composite of experiments XI–XIII (a, b), XV–XVII (c), XIV–XVII (d), XIV, XV, XVII (e) and XVIII, XIX (f). (PDF 243 kb)
Additional file 8: Figure S8. SKN-1 and DAF-16 target gene expression upon *impt-1* RNAi. (a) Average integrated density of GFP expression of the *Pgst-4::GFP* reporter at different larval stages [L1 (L4440, *n* = 19; *impt-1*, *n* = 20), L2 (L4440, *n* = 36; *impt-1*, *n* = 29), L4 (L4440, *n* = 23; *impt-1*, *n* = 31); Student *t* test). This is a composite of two independent experiments. (b) Representative images of *Pgst-4::GFP* expression in L2 larvae treated with L4440 (control) or *impt-1* RNAi from L1. Dashed lines delimitate the body of the worm. Head and tail are indicated by arrows. (c) Average integrated density of GFP expression of the *Psod-3::GFP* reporter at different larval stages [L1 (L4440, *n* = 9; *impt-1*, *n* = 12), L2 (L4440, *n* = 18; *impt-1*, *n* = 24), L4 (L4440, *n* = 14; *impt-1*, *n* = 12); **P* < 0.05, Two-way ANOVA, Sidak *post-hoc*]. This is composite of two independent experiments. Bars are mean ± SEM. (PDF 390 kb)
Additional file 9: Figure S9.
*Impt-1* expression in response to DR or aging. (a) *Impt-1* mRNA expression in N2 worms and worms carrying the *daf-16(m26)*, *skn-1(zu135)*, and *hsf-1(sy441)* mutations. These genes are necessary for some protocols of DR to extend lifespan in *C. elegans*. (b) *Impt-1* mRNA expression in N2 worms and *eat-2* (DR model) on days 0 and 10. *n* = 3 pools of at least 150 worms per group. Data are presented as mean ± SEM and compared using a one-way ANOVA with Dunnet post-hoc (a) or two-way ANOVA (b). The experiments were performed once in triplicate. **P* < 0.05 versus day 0. Raw data in Additional file 12: Table S3. (PDF 59 kb)
Additional file 10: Table S1. Compilation of lifespan data. (DOCX 51 kb)
Additional file 11: Table S2. qPCR data of Additional file 2: Figure S2. (XLSX 12 kb)
Additional file 12: Table S3. qPCR data of Additional file 9: Figure S9. (XLSX 14 kb)
