# Supplementary material for: IMPACT is a GCN2 inhibitor that limits lifespan in Caenorhabditis elegans
Source: BMC Biol. 2016 Oct 7;14:87. doi: 10.1186/s12915-016-0301-2 (PMC5054600; doi:10.1186/s12915-016-0301-2)
Supplement: Additional file 1: Figure S1. — The IMPACT protein of Caenorhabditis elegans. (a) Alignment between the amino acid sequences of mouse protein IMPACT (sbjct) and Y52B11A.2 of C. elegans (query). Result show 33 % identity and 51 % similarity between both proteins (E value of 7e–55). (b) Predicted domains of C. elegans Y52B11A.2. (PDF 270 kb) [file 12915_2016_301_MOESM1_ESM.pdf]

a

Range 1: 6 to 293 GenPept Graphics ▼ Next Match ▲ Previous Match

Score	Expect	Method	Identities	Positives	Gaps
167 bits(422)	7e-55	Compositional matrix adjust.	95/291(33%)	150/291(51%)	42/291(14%)

```
Query  1    MSDEEQRIAEIESLASIFPDLLEETTDKQLVFKF---------DRDIGMTINLPEDYPSI  51
            + + +++  EIE++A+I+ +      +   +F              + + + LP +YP
Sbjct  6    VGNSQRQSEEIEAMAAIYGEEWCVIDENAKIFCIRVTDFMDDPKWTLCLQVMLPSEYPGT  65

Query  52   SPPIFELSGPYLRREQKEVLHNSLNDVYIENIGFPVIFNWISLVQDFIRDLPEEVAEP--  109
            +PP ++L+ P+L+ +++  L NSL ++Y+ N+G  +++ W+  ++D +     ++ EP
Sbjct  66   APPSYQLNAPWLKGQERADLSNSLEEIYVHNMGESILYQWVEKIRDALIQ-KSQITEPDP  124

Query  110  -------------------SHVADDVVTPMAEEIDDMN---------IRHGEVLTDRKSA  141
                                H  ++ V  +  +I +           + HG  +TDR+S
Sbjct  125  DVKKKTEEVEVESEEDPILEHPPENPVKTLDLKISEETQPETEELPPVAHGVPITDRRST  184

Query  142  FQAHLAEVRSKEDVDRVMRILKSNTKICRATHNITAYRYMTEINGKPIHHHDCVDDGEFG  201
            FQAH+A V   E V  V+  L  N KI  ATHNI AYR   E   K     DC DDGE
Sbjct  185  FQAHVAPVVCPEQVKLVLAKLYENKKIASATHNIYAYRIFCE--DKQTFLQDCEDDGETA  242

Query  202  ASSKMLELMDRMKADNVMVVVSRWYGGIHLGPDRFRHINNLTRQILSDNNY  252
            A  ++L LM+ +   NVMVVVSRWYGGI LGPDRF+HINN  R IL + N+
Sbjct  243  AGGRLLHLMEILNVKNVMVVVSRWYGGILLGPDRFKHINNCARNILVEKNF  293
```

b

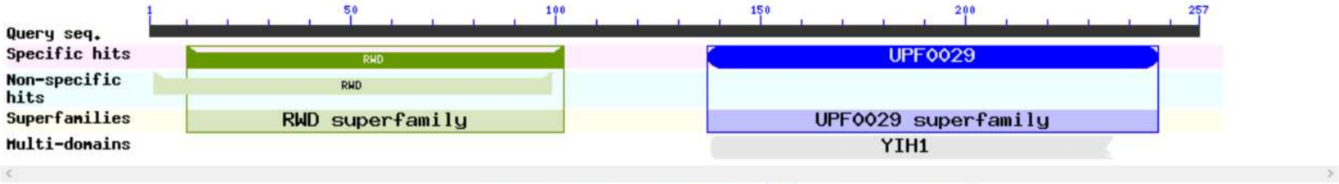

Search for similar domain architectures | Refine search

List of domain hits

Name	Accession	Description	Interval	E-value
[+] UPF0029	pfam01205	Uncharacterized protein family UPF0029;	137-247	6.42e-36
[+] RWD	smart00591	domain in RING finger and WD repeat containing proteins and DEXDc-like helicases subfamily ...	10-102	7.62e-21
[+] RWD	pfam05773	RWD domain; This domain was identified in WD40 repeat proteins and Ring finger domain proteins. ...	2-99	7.88e-18
[+] YIH1	COG1739	Putative translation regulator, IMPACT (imprinted ancient) protein family [General function ...	138-236	2.08e-07
