# Supplementary figures and images for: IMPACT is a GCN2 inhibitor that limits lifespan in Caenorhabditis elegans
Source: BMC Biol. 2016 Oct 7;14:87. doi: 10.1186/s12915-016-0301-2 (PMC5054600; doi:10.1186/s12915-016-0301-2)

**a**

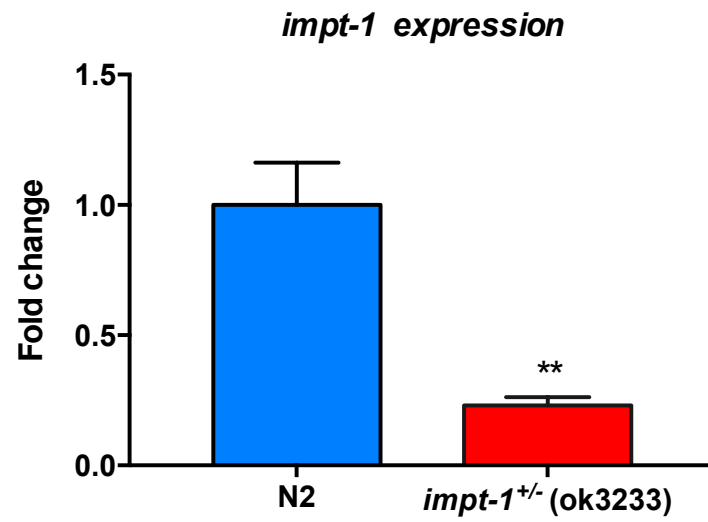

**b**

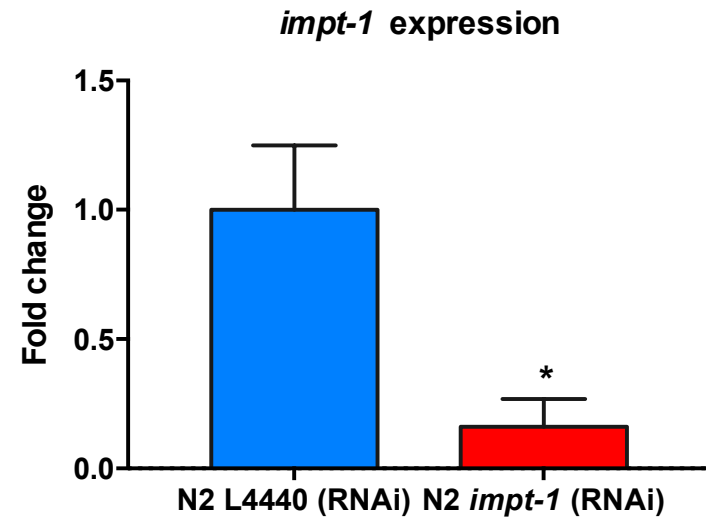

Supplement: Additional file 2: Figure S2. — Impt-1 expression in impt-1 +/– mutants and worms exposed to impt-1 RNAi. (a,b) Impt-1 mRNA expression in day 0 N2 (n = 3 pools of 150 worms) and impt-1 +/– (ok3233) (n = 3 pools of 150 worms) worms (a) or N2 worms treated with L4440 (control) (n = 3 pools of 150 worms) or impt-1 RNAi (n = 3 pools of 150 worms) from eggs (b). Data are presented as mean ± SEM and compared using unpaired Student t test, **P = 0.0097 in comparison to N2, *P = 0.036 in comparison to L4440. The experiments were performed once in triplicate. Raw data in Additional file 11: Table S2. (PDF 296 kb) [file 12915_2016_301_MOESM2_ESM.pdf]

**a**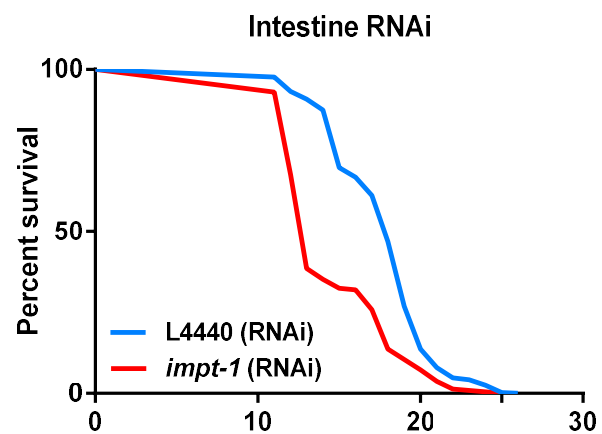**b**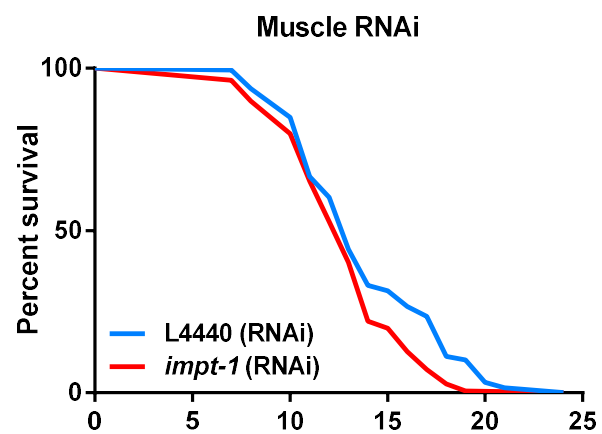**c**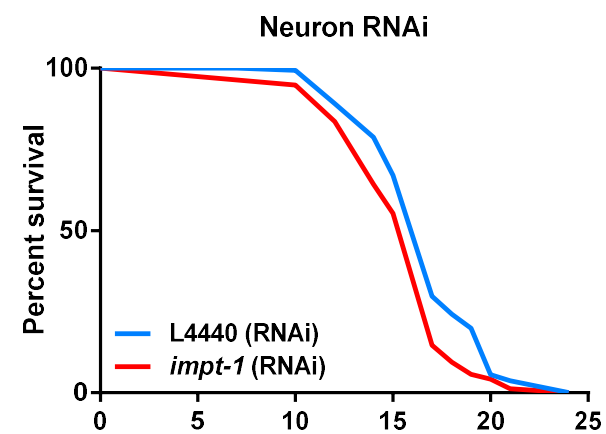

Supplement: Additional file 3: Figure S3. — Tissue-specific impt-1 RNAi in intestine, muscle, or neurons is not sufficient to increase lifespan. (a–c) Lifespan assays of worms with tissue-specific RNAi sensitivity in intestine (a), muscle (b), or neurons (c). The worms were treated with L4440 (control) or impt-1 RNAi from L1. Values of median lifespan and statistics are reported in Additional file 10: Table S1. Survival curves were compared using the long-rank test. All experiments were repeated three times. Data demonstrate the composite of experiments XXIII–XXV (a), XXVI–XXVIII (b), or XXIX–XXXI (c). (PDF 52 kb) [file 12915_2016_301_MOESM3_ESM.pdf]

**a**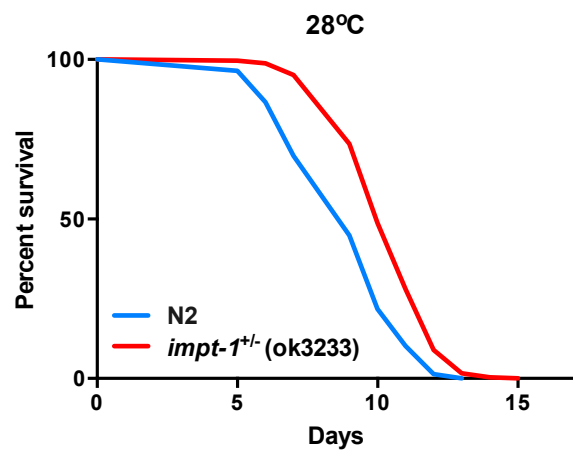**b**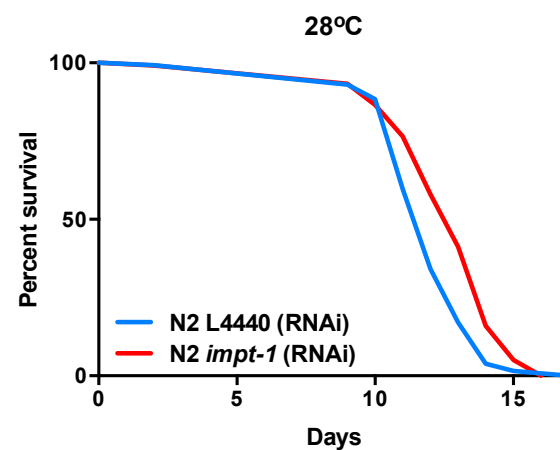**c**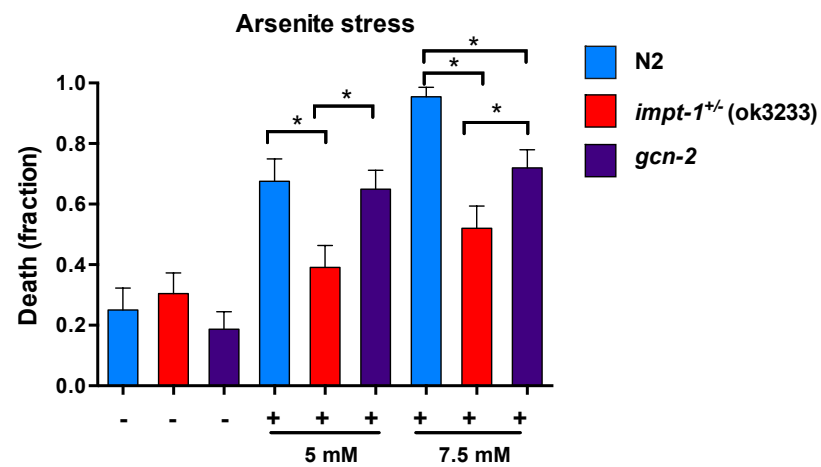**d**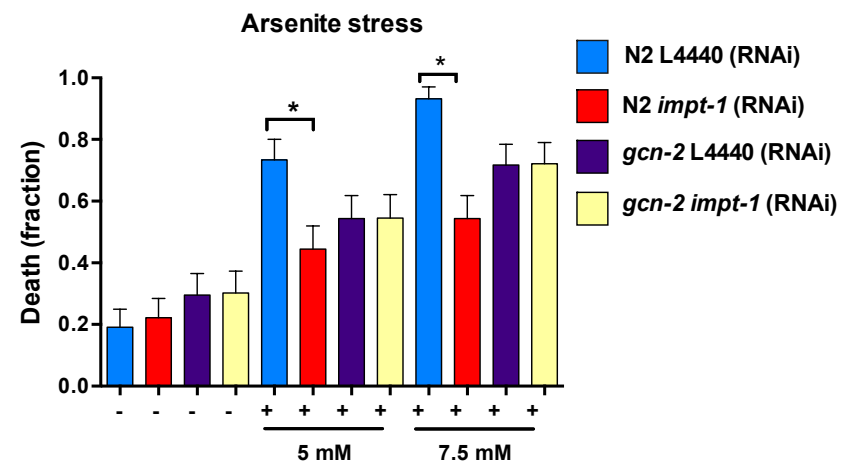

Supplement: Additional file 4: Figure S4. — Impt-1 knockdown promotes stress resistance. (a, b) Lifespan assays under heat stress (28 °C) of N2 worms and impt-1 +/– (ok3233) mutants (a) or N2 worms treated with L4440 (control) or impt-1 RNAi from L1 (b). Values of median lifespan and statistics are reported in Additional file 10: Table S1. Survival curves were compared by log-rank test. Experiment (a) was repeated three times and (b) was performed once. Data demonstrate the composite of experiments XXXII–XXXIV (a) or the experiment XXXV (b). (c) Fraction of dead N2, impt-1 +/– (ok3233) and gcn-2(ok871) worms when exposed to 5 mM or 7.5 mM sodium arsenite for 7 hours (N2, n = 44; impt-1 +/–, n = 46; gcn-2, n = 48; *P < 0.05; One-way ANOVA, Tukey post-hoc). This is a representative experiment of two independent experiments. (d) Fraction of dead worms after treatment with 5 mM or 7.5 mM sodium arsenite for 7 hours. This is a representative experiment of two independent experiments (N2 L4440 RNAi, n = 47; N2 impt-1 RNAi, n = 46; gcn-2 L4440 RNAi, n = 46; gcn-2 impt-1 RNAi, n = 44; *P < 0.05; Two-way ANOVA, Tukey post-hoc). RNAi was initiated at L1. Data are presented as mean ± SEM. (PDF 303 kb) [file 12915_2016_301_MOESM4_ESM.pdf]

**a**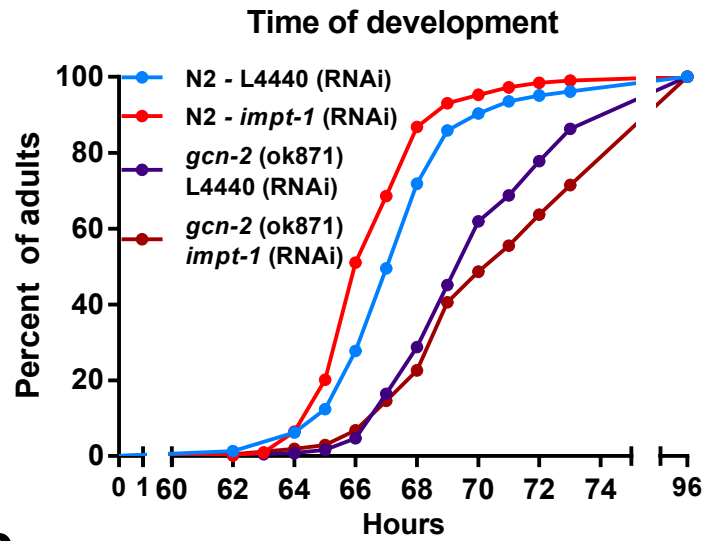**b**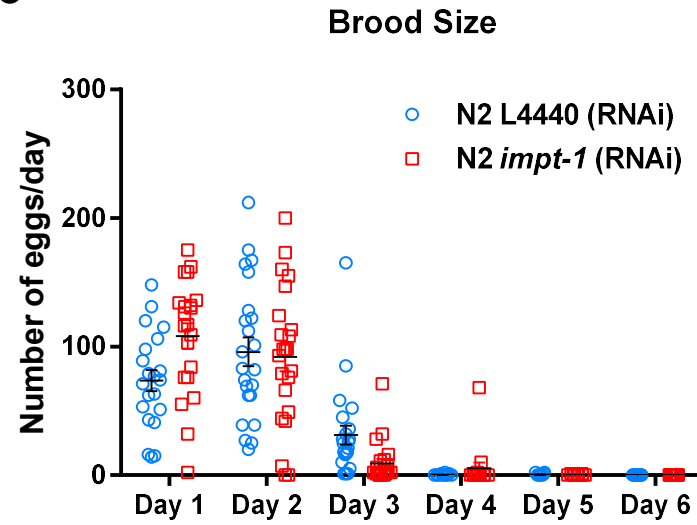**c**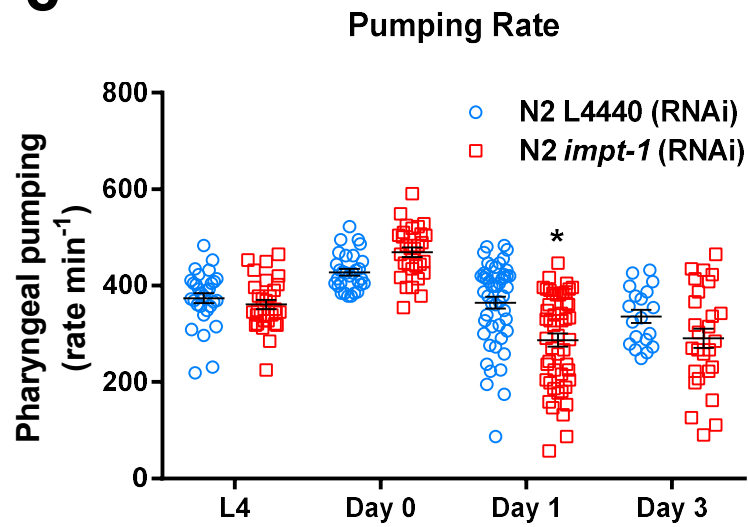**d**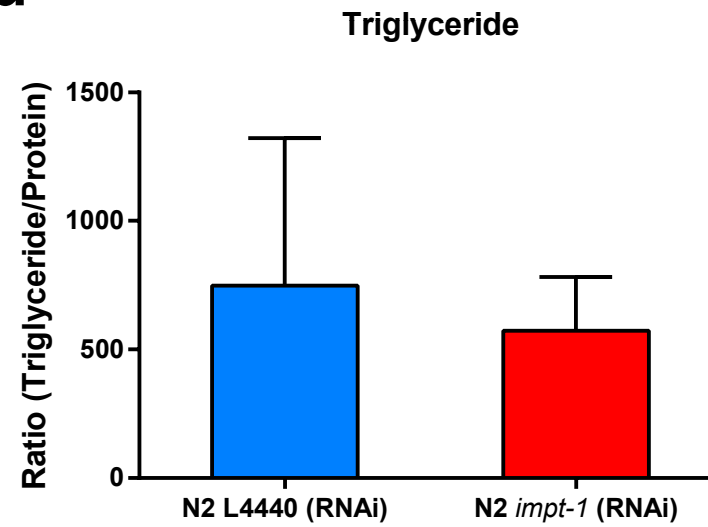

Supplement: Additional file 5: Figure S5. — Phenotypic characterization of N2 worms treated with impt-1 RNAi from L1. (a) Developmental timing after egg laying of N2 and gcn-2(ok871) worms (N2 L4440 RNAi, n = 469; N2 impt-1 RNAi, n = 510; gcn-2 L4440 RNAi, n = 365; gcn-2 impt-1 RNAi, n = 411; P < 0.05; log-rank test; all groups are statistically different compared to each other). This is the composite of three experiments. (b) Brood size (N2 L4440 RNAi, n = 24; N2 impt-1 RNAi, n = 24 *P < 0.05, Two-way ANOVA, Bonferroni post-hoc). This is a composite of three independent experiments. (c) Pharyngeal pumping rate at different ages [L4 (L4440, n = 30; impt-1, n = 30), Day 0 (L4440, n = 30; impt-1, n = 30), Day 1 (L4440, n = 50; impt-1, n = 50), Day 3 (L4440, n = 19; impt-1, n = 26); *P < 0.05, Two-way ANOVA, Sidak post-hoc)]. Each time point was scored at least twice and the data is the composite of five experiments. (d) Triglyceride levels at day 1 of adulthood normalized by protein levels (n = 4 pools of at least 150 worms; unpaired Student t test). Bars are presented as mean ± SEM. Each pool was obtained in independent experiments. (PDF 274 kb) [file 12915_2016_301_MOESM5_ESM.pdf]

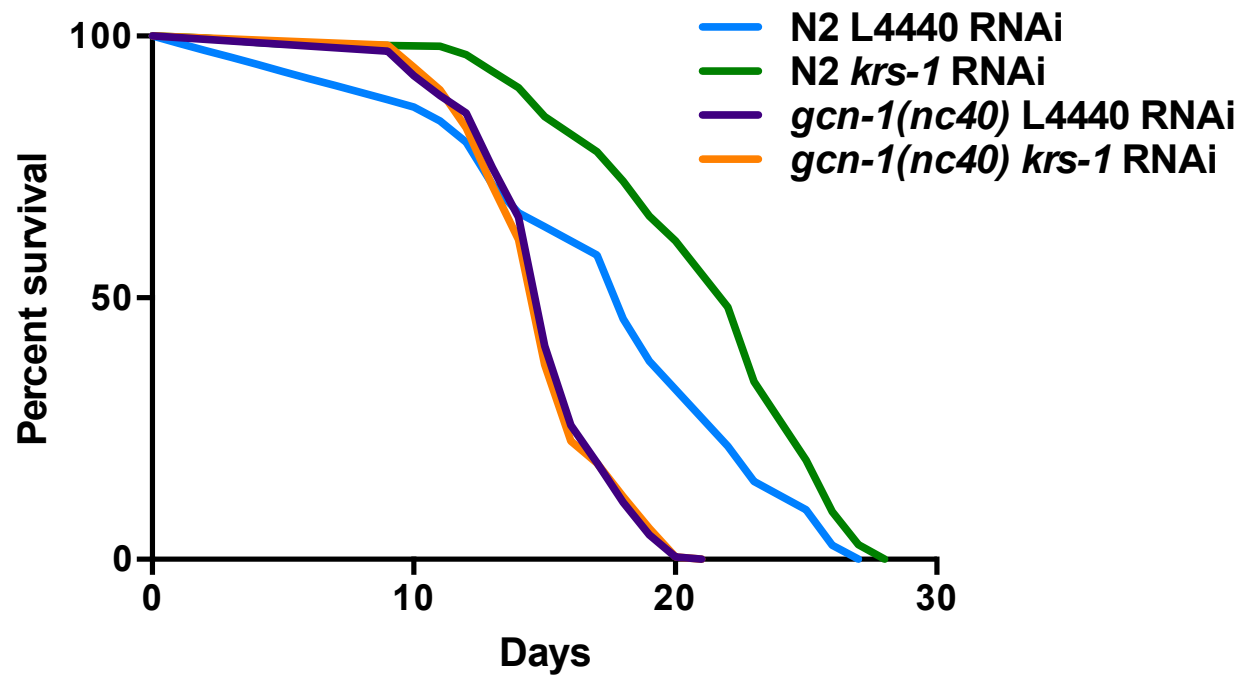

Supplement: Additional file 6: Figure S6. — Lifespan increase induced by krs-1 RNAi is blocked in gcn-1 mutants. Lifespan assay of N2 worms and gcn-1(nc40) mutants treated with L4440 or krs-1 RNAi from day 0 adults. Values of median lifespan and statistics are reported in Additional file 10: Table S1. Survival curves were compared using the log-rank test. Data demonstrate the composite of experiments XXXVI and XXXVII. (PDF 146 kb) [file 12915_2016_301_MOESM6_ESM.pdf]

**a**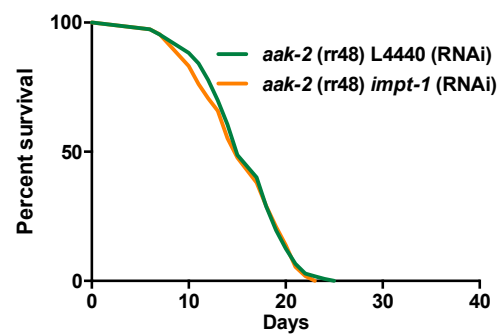**b**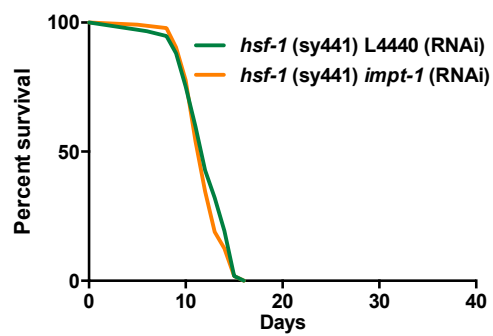**c**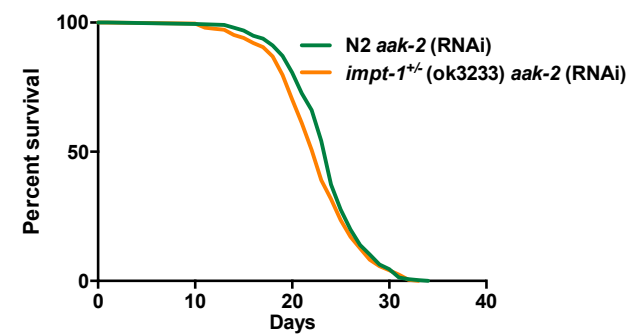**d**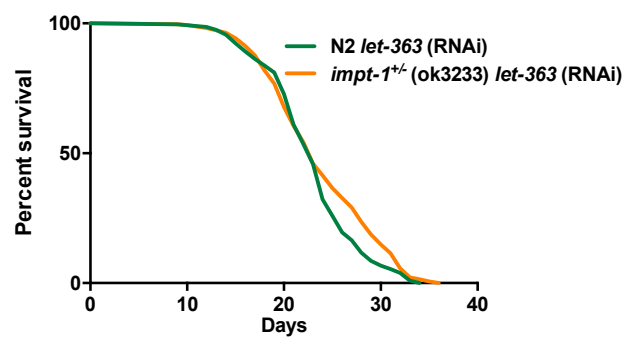**e**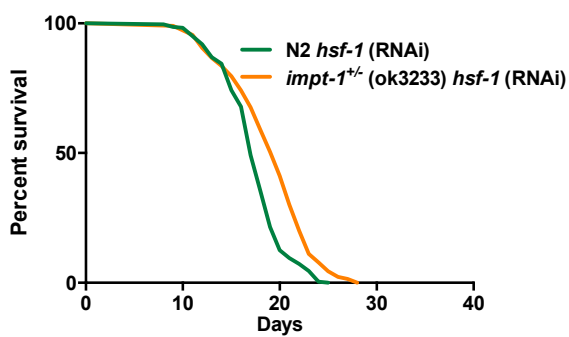**f**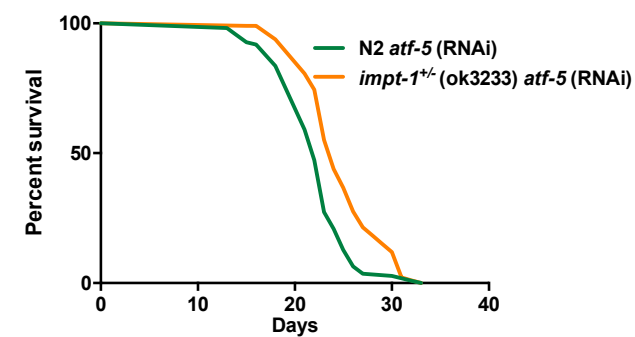

Supplement: Additional file 7: Figure S7. — Impt-1 interactions with components of the DR pathway to control longevity. (a,b) Lifespan assays of aak-2(n48) and hsf-1(sy441) mutants treated with L4440 (control) or impt-1 RNAi from L1. (c–f) Lifespan assays of N2 worms and impt-1 +/– (ok3233) mutants treated with aak-2, let-363, hsf-1, or atf-5 RNAi from day 0 adults. Values of median lifespan and statistics are reported in Additional file 10: Table S1. Survival curves were compared using the log-rank test. All experiments were repeated at least twice. Data demonstrate the composite of experiments XI–XIII (a, b), XV–XVII (c), XIV–XVII (d), XIV, XV, XVII (e) and XVIII, XIX (f). (PDF 243 kb) [file 12915_2016_301_MOESM7_ESM.pdf]

**a**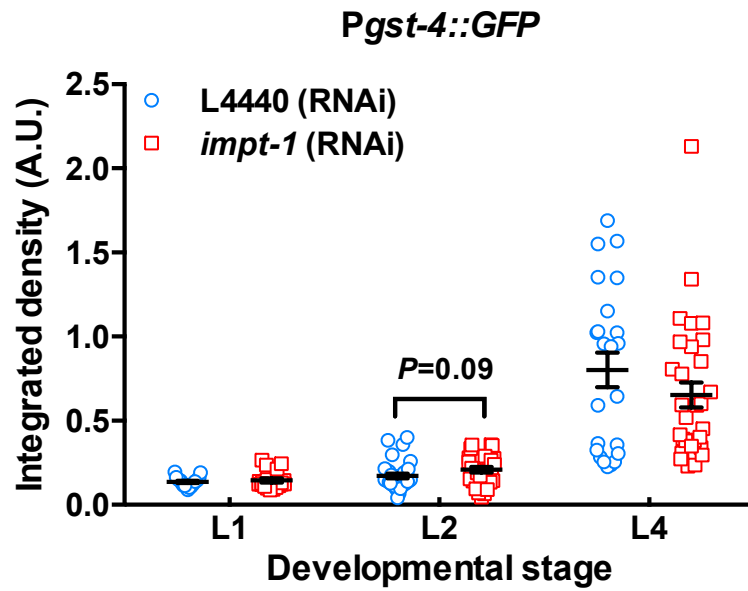**b**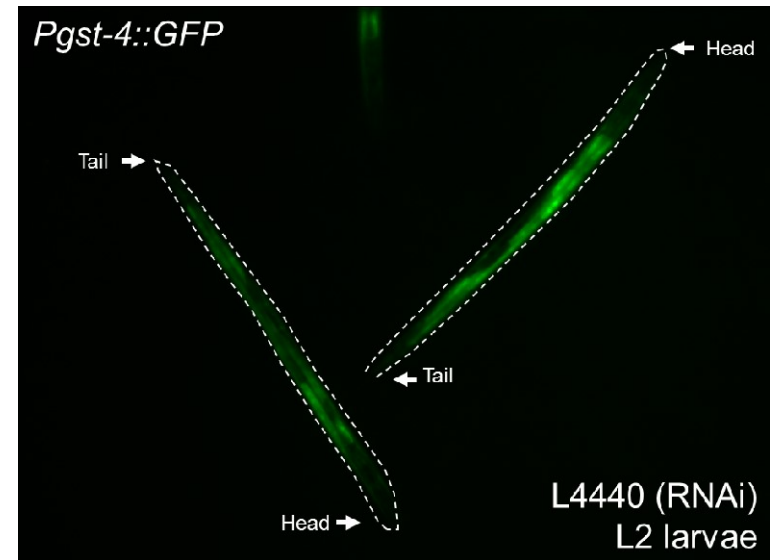**c**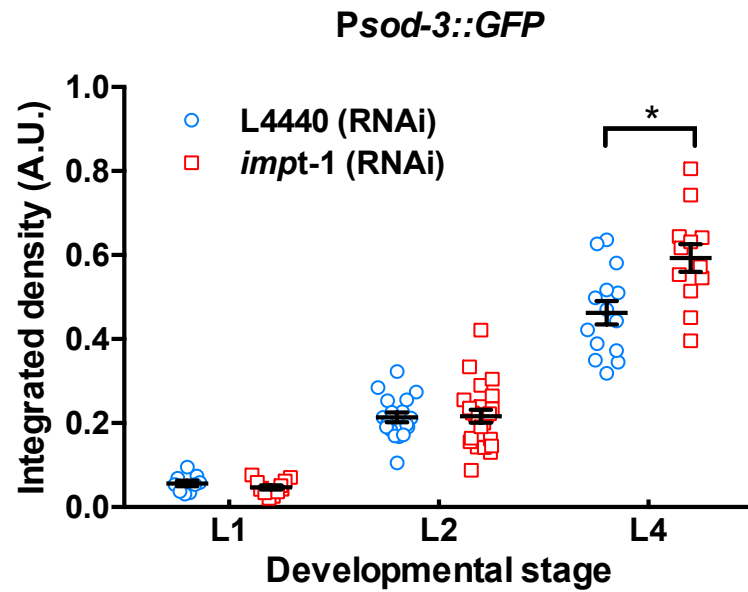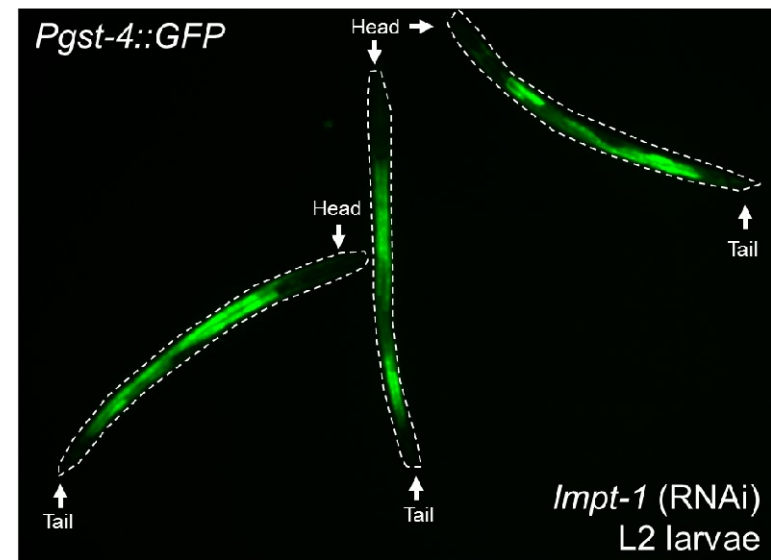

Supplement: Additional file 8: Figure S8. — SKN-1 and DAF-16 target gene expression upon impt-1 RNAi. (a) Average integrated density of GFP expression of the Pgst-4::GFP reporter at different larval stages [L1 (L4440, n = 19; impt-1, n = 20), L2 (L4440, n = 36; impt-1, n = 29), L4 (L4440, n = 23; impt-1, n = 31); Student t test). This is a composite of two independent experiments. (b) Representative images of Pgst-4::GFP expression in L2 larvae treated with L4440 (control) or impt-1 RNAi from L1. Dashed lines delimitate the body of the worm. Head and tail are indicated by arrows. (c) Average integrated density of GFP expression of the Psod-3::GFP reporter at different larval stages [L1 (L4440, n = 9; impt-1, n = 12), L2 (L4440, n = 18; impt-1, n = 24), L4 (L4440, n = 14; impt-1, n = 12); *P < 0.05, Two-way ANOVA, Sidak post-hoc]. This is composite of two independent experiments. Bars are mean ± SEM. (PDF 390 kb) [file 12915_2016_301_MOESM8_ESM.pdf]

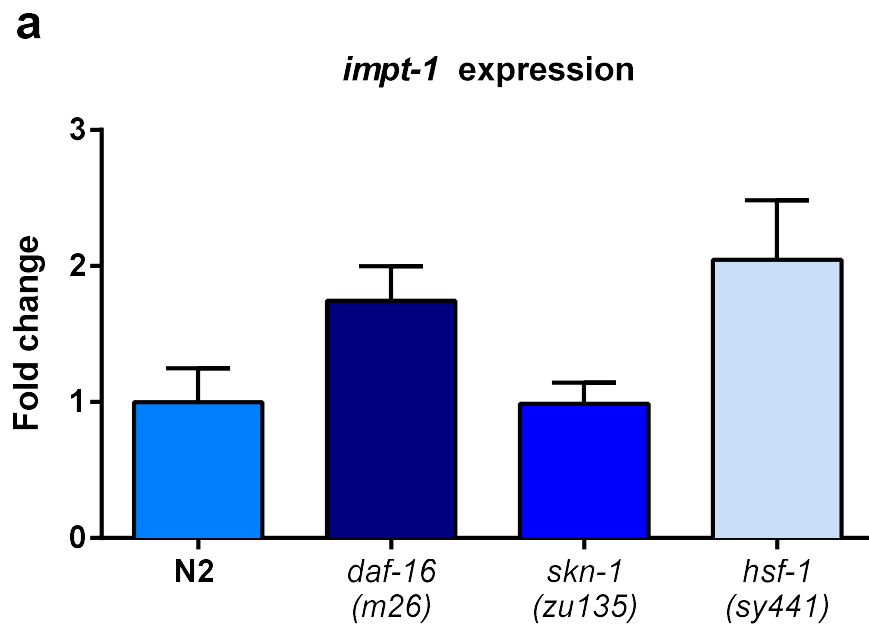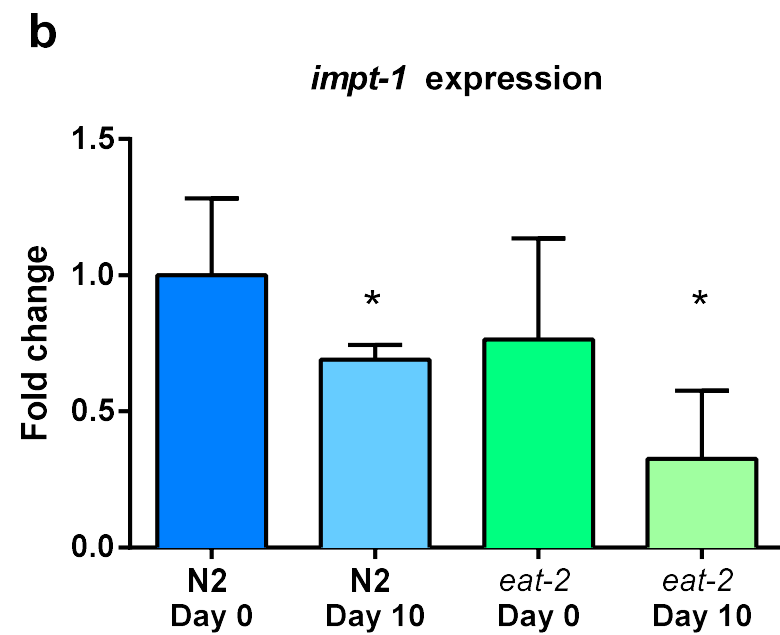

Supplement: Additional file 9: Figure S9. — Impt-1 expression in response to DR or aging. (a) Impt-1 mRNA expression in N2 worms and worms carrying the daf-16(m26), skn-1(zu135), and hsf-1(sy441) mutations. These genes are necessary for some protocols of DR to extend lifespan in C. elegans. (b) Impt-1 mRNA expression in N2 worms and eat-2 (DR model) on days 0 and 10. n = 3 pools of at least 150 worms per group. Data are presented as mean ± SEM and compared using a one-way ANOVA with Dunnet post-hoc (a) or two-way ANOVA (b). The experiments were performed once in triplicate. *P < 0.05 versus day 0. Raw data in Additional file 12: Table S3. (PDF 59 kb) [file 12915_2016_301_MOESM9_ESM.pdf]
