# Supplementary material for: IMPACT is a GCN2 inhibitor that limits lifespan in Caenorhabditis elegans
Source: BMC Biol. 2016 Oct 7;14:87. doi: 10.1186/s12915-016-0301-2 (PMC5054600; doi:10.1186/s12915-016-0301-2)
Supplement: Additional file 10: Table S1. — Compilation of lifespan data. (DOCX 51 kb) [file 12915_2016_301_MOESM10_ESM.docx]

**Supplementary Table 1| Compilation of lifespan data.**

| **Experiment** | **Strain** | ***Allele*** | **Temperature (°C)** | **Condition** | **Median Lifespan** | **N** | **Median Lifespan Effect** | ***P* value vs. control** |
| --- | --- | --- | --- | --- | --- | --- | --- | --- |
| I | N2 | *-* | 20 | *-* | 23 | 111 | 27.78% | <0.0001 |
| I | VC2511 | *Y52B11A.2(ok3233) I/hT2 [bli-4(e937) let-?(q782) qIs48] (I;III)* | 20 | *-* | 18 | 80 | - | - |
| II | N2 | *-* | 20 | *impt-1 RNAi* | 20 | 116 | 11.11% | <0.0001 |
| II | N2 | *-* | 20 | *L4440 RNAi* | 18 | 80 | - | - |
| II | RB976 | *Y81G3A.3(ok871) II.* | 20 | *impt-1 RNAi* | 20 | 83 | 0% | - |
| II | RB976 | *Y81G3A.3(ok871) II.* | 20 | *L4440 RNAi* | 20 | 84 | - | - |
| II | ST60 | *gcn-1(nc40) III.* | 20 | *impt-1 RNAi* | 17 | 136 | 0% | - |
| II | ST60 | *gcn-1(nc40) III.* | 20 | *L4440 RNAi* | 17 | 155 | - | - |
| III | N2 | *-* | 20 | *impt-1 RNAi* | 22 | 173 | 15.79% | 0.0222 |
| III | N2 | *-* | 20 | *L4440 RNAi* | 19 | 83 | - | - |
| III | RB976 | *Y81G3A.3(ok871) II.* | 20 | *impt-1 RNAi* | 20 | 118 | 0% | - |
| III | RB976 | *Y81G3A.3(ok871) II.* | 20 | *L4440 RNAi* | 20 | 102 | - | - |
| III | ST60 | *gcn-1(nc40) III.* | 20 | *impt-1 RNAi* | 16 | 119 | -5.88% | - |
| III | ST60 | *gcn-1(nc40) III.* | 20 | *L4440 RNAi* | 17 | 131 | - | - |
| IV | N2 | *-* | 20 | *impt-1 RNAi* | 23 | 196 | 21.05% | 0.0054 |
| IV | N2 | *-* | 20 | *L4440 RNAi* | 19 | 83 | - | - |
| IV | RB976 | *Y81G3A.3(ok871) II.* | 20 | *impt-1 RNAi* | 22 | 106 | 0% | - |
| IV | RB976 | *Y81G3A.3(ok871) II.* | 20 | *L4440 RNAi* | 22 | 109 | - | - |
| V | N2 | *(tm4397)background* | 20 | *impt-1 RNAi* | 25 | 95 | 8% | 0.0073 |
| V | N2 | *(tm4397)background* | 20 | *L4440 RNAi* | 23 | 80 |  |  |
| V | atf-5 | *atf-5(tm4397) X.* | 20 | *impt-1 RNAi* | 24 | 112 | 0% | - |
| V | atf-5 | *atf-5(tm4397) X.* | 20 | *L4440 RNAi* | 24 | 122 | - | - |
| VI | N2 | *(tm4397)background* | 20 | *impt-1 RNAi* | 26 | 104 | 13% | 0.0019 |
| VI | N2 | *(tm4397)background* | 20 | *L4440 RNAi* | 23 | 95 | - |  |
| VI | atf-5 | *atf-5(tm4397) X.* | 20 | *impt-1 RNAi* | 23 | 121 | 0% | - |
| VI | atf-5 | *atf-5(tm4397) X.* | 20 | *L4440 RNAi* | 23 | 115 | - | - |
| VII | DA1116 | *eat-2(ad1116) II.* | 20 | *impt-1 RNAi* | 24 | 96 | 9.09% | 0.0044 |
| VII | DA1116 | *eat-2(ad1116) II.* | 20 | *L4440 RNAi* | 22 | 95 | - | - |
| VIII | DA1116 | *eat-2(ad1116) II.* | 20 | *impt-1 RNAi* | 25 | 125 | 25% | 0.0001 |
| VIII | DA1116 | *eat-2(ad1116) II.* | 20 | *L4440 RNAi* | 20 | 89 | - | - |
| IX | N2 | *-* | 20 | *krs-1 RNAi* | 23 | 255 | 21.05% | 0.0001 |
| IX | N2 | *-* | 20 | *L4440 RNAi* | 19 | 83 | - | - |
| IX | VC2511 | *Y52B11A.2(ok3233) I/hT2 [bli-4(e937) let-?(q782) qIs48] (I;III)* | 20 | *krs-1 RNAi* | 25 | 217 | 8.7% | 0.0001 |
| IX | VC2511 | *Y52B11A.2(ok3233) I/hT2 [bli-4(e937) let-?(q782) qIs48] (I;III)* | 20 | *L4440 RNAi* | 23 | 196 | - | - |
| X | N2 | *-* | 20 | *krs-1 RNAi* | 24 | 115 | 26.32% | 0.0001 |
| X | N2 | *-* | 20 | *L4440 RNAi* | 19 | 96 | - | - |
| X | VC2511 | *Y52B11A.2(ok3233) I/hT2 [bli-4(e937) let-?(q782) qIs48] (I;III)* | 20 | *krs-1 RNAi* | 25 | 172 | 8.7% | 0.0001 |
| X | VC2511 | *Y52B11A.2(ok3233) I/hT2 [bli-4(e937) let-?(q782) qIs48] (I;III))* | 20 | *L4440 RNAi* | 23 | 142 | - | - |
| XI | N2 | *-* | 20 | *impt-1 RNAi* | 20 | 113 | 11.11% | 0.0001 |
| XI | N2 | *-* | 20 | *L4440 RNAi* | 18 | 83 | - | - |
| XI | MR507 | *aak-2(rr48) X.* | 20 | *impt-1 RNAi* | 14 | 109 | -6.67% | - |
| XI | MR507 | *aak-2(rr48) X.* | 20 | *L4440 RNAi* | 15 | 161 | - | - |
| XI | DR26 | *daf-16(m26) I.* | 20 | *impt-1 RNAi* | 15 | 127 | 3.45% | - |
| XI | DR26 | *daf-16(m26) I.* | 20 | *L4440 RNAi* | 14.5 | 128 | - | - |
| XI | PS3551 | *hsf-1(sy441) I.* | 20 | *impt-1 RNAi* | 12 | 137 | 0% | - |
| XI | PS3551 | *hsf-1(sy441) I.* | 20 | *L4440 RNAi* | 12 | 97 | - | - |
| XI | EU31 | *skn-1(zu135)* | 20 | *impt-1 RNAi* | 13 | 65 | 0% | - |
| XI | EU31 | *skn-1(zu135)* | 20 | *L4440 RNAi* | 13 | 116 | - | - |
| XII | N2 | *-* | 20 | *impt-1 RNAi* | 22 | 96 | 15.79% | 0.043 |
| XII | N2 | *-* | 20 | *L4440 RNAi* | 19 | 134 | - | - |
| XII | MR507 | *aak-2(rr48) X.* | 20 | *impt-1 RNAi* | 14 | 107 | -6.67% | - |
| XII | MR507 | *aak-2(rr48) X.* | 20 | *L4440 RNAi* | 15 | 142 | - | - |
| XII | DR26 | *daf-16(m26) I.* | 20 | *impt-1 RNAi* | 17 | 123 | 13.33% | - |
| XII | DR26 | *daf-16(m26) I.* | 20 | *L4440 RNAi* | 15 | 138 | - | - |
| XII | PS3551 | *hsf-1(sy441) I.* | 20 | *impt-1 RNAi* | 12 | 123 | 0% | - |
| XII | PS3551 | *hsf-1(sy441) I.* | 20 | *L4440 RNAi* | 12 | 109 | - | - |
| XII | EU31 | *skn-1(zu135)* | 20 | *impt-1 RNAi* | 14 | 53 | -6.67% | - |
| XII | EU31 | *skn-1(zu135)* | 20 | *L4440 RNAi* | 15 | 114 | - | - |
| XIII | N2 | *-* | 20 | *impt-1 RNAi* | 22 | 155 | 18.92% | 0.0012 |
| XIII | N2 | *-* | 20 | *L4440 RNAi* | 18.5 | 86 | - | - |
| XIII | MR507 | *aak-2(rr48) X.* | 20 | *impt-1 RNAi* | 16 | 94 | 0% | - |
| XIII | MR507 | *aak-2(rr48) X.* | 20 | *L4440 RNAi* | 16 | 89 | - | - |
| XIII | DR26 | *daf-16(m26) I.* | 20 | *impt-1 RNAi* | 13 | 70 | 0% | - |
| XIII | DR26 | *daf-16(m26) I.* | 20 | *L4440 RNAi* | 13 | 138 | - | - |
| XIII | PS3551 | *hsf-1(sy441) I.* | 20 | *impt-1 RNAi* | 12 | 116 | 0% | - |
| XIII | PS3551 | *hsf-1(sy441) I.* | 20 | *L4440 RNAi* | 12 | 121 | - | - |
| XIII | EU31 | *skn-1(zu135)* | 20 | *impt-1 RNAi* | 14 | 155 | 7.69% | - |
| XIII | EU31 | *skn-1(zu135)* | 20 | *L4440 RNAi* | 13 | 80 | - | - |
| XIV | N2 | *-* | 20 | *daf-16 RNAi* | 13 | 31 | - |  |
| XIV | VC2511 | *Y52B11A.2(ok3233) I/hT2 [bli-4(e937) let-?(q782) qIs48] (I;III)* | 20 | *daf-16 RNAi* | 12 | 37 | -7.69% | 0.0058 |
| XIV | N2 | *-* | 20 | *let-363 RNAi* | 22 | 127 | - |  |
| XIV | VC2511 | *Y52B11A.2(ok3233) I/hT2 [bli-4(e937) let-?(q782) qIs48] (I;III)* | 20 | *let-363 RNAi* | 20 | 40 | -9.09% | < 0.0001 |
| XIV | N2 | *-* | 20 | *hsf-1 RNAi* | 13 | 54 | - |  |
| XIV | VC2511 | *Y52B11A.2(ok3233) I/hT2 [bli-4(e937) let-?(q782) qIs48] (I;III)* | 20 | *hsf-1 RNAi* | 14 | 45 | 7.69% | 0.798 |
| XV | N2 | *-* | 20 | *L4440* | 20 | 274 | - |  |
| XV | VC2511 | *Y52B11A.2(ok3233) I/hT2 [bli-4(e937) let-?(q782) qIs48] (I;III)* | 20 | *L4440* | 23 | 121 | 15% | < 0.0001 |
| XV | N2 | *-* | 20 | *daf-16 RNAi* | 18 | 145 | - |  |
| XV | VC2511 | *Y52B11A.2(ok3233) I/hT2 [bli-4(e937) let-?(q782) qIs48] (I;III)* | 20 | *daf-16 RNAi* | 18 | 110 | 0% | 0.281 |
| XV | N2 | *-* | 20 | *let-363 RNAi* | 26 | 112 | - |  |
| XV | VC2511 | *Y52B11A.2(ok3233) I/hT2 [bli-4(e937) let-?(q782) qIs48] (I;III)* | 20 | *let-363 RNAi* | 28 | 148 | 7.69% | 0.1037 |
| XV | N2 | *-* | 20 | *aak-2 RNAi* | 24 | 117 | - |  |
| XV | VC2511 | *Y52B11A.2(ok3233) I/hT2 [bli-4(e937) let-?(q782) qIs48] (I;III)* | 20 | *aak-2 RNAi* | 23 | 105 | -4.17% | 0.2687 |
| XV | N2 | *-* | 20 | *hsf-1 RNAi* | 19 | 92 | - |  |
| XV | VC2511 | *Y52B11A.2(ok3233) I/hT2 [bli-4(e937) let-?(q782) qIs48] (I;III)* | 20 | *hsf-1 RNAi* | 21 | 115 | 10.53% | 0.0117 |
| XVI | N2 | *-* | 20 | *L4440* | 25 | 164 | - |  |
| XVI | VC2511 | *Y52B11A.2(ok3233) I/hT2 [bli-4(e937) let-?(q782) qIs48] (I;III)* | 20 | *L4440* | 22 | 138 | -12% | 0.0839 |
| XVI | N2 | *-* | 20 | *daf-16 RNAi* | 15 | 140 | - |  |
| XVI | VC2511 | *Y52B11A.2(ok3233) I/hT2 [bli-4(e937) let-?(q782) qIs48] (I;III)* | 20 | *daf-16 RNAi* | 14 | 119 | -6.67% | < 0.0001 |
| XVI | N2 | *-* | 20 | *let-363 RNAi* | 21 | 179 | - |  |
| XVI | VC2511 | *Y52B11A.2(ok3233) I/hT2 [bli-4(e937) let-?(q782) qIs48] (I;III)* | 20 | *let-363 RNAi* | 20 | 118 | -4.76% | 0.5074 |
| XVI | N2 | *-* | 20 | *aak-2 RNAi* | 24 | 169 | - |  |
| XVI | VC2511 | *Y52B11A.2(ok3233) I/hT2 [bli-4(e937) let-?(q782) qIs48] (I;III)* | 20 | *aak-2 RNAi* | 23 | 103 | -4.17% | 0.005 |
| XVII | N2 | *-* | 20 | *L4440* | 23 | 126 | - |  |
| XVII | VC2511 | *Y52B11A.2(ok3233) I/hT2 [bli-4(e937) let-?(q782) qIs48] (I;III)* | 20 | *L4440* | 24 | 86 | 4.35% | 0.017 |
| XVII | N2 | *-* | 20 | *daf-16 RNAi* | 17 | 215 | - |  |
| XVII | VC2511 | *Y52B11A.2(ok3233) I/hT2 [bli-4(e937) let-?(q782) qIs48] (I;III)* | 20 | *daf-16 RNAi* | 16 | 94 | -5.88% | 0.0061 |
| XVII | N2 | *-* | 20 | *let-363 RNAi* | 26 | 69 | - |  |
| XVII | VC2511 | *Y52B11A.2(ok3233) I/hT2 [bli-4(e937) let-?(q782) qIs48] (I;III)* | 20 | *let-363 RNAi* | 23 | 30 | -11.54% | 0.6368 |
| XVII | N2 | *-* | 20 | *aak-2 RNAi* | 23 | 74 | - |  |
| XVII | VC2511 | *Y52B11A.2(ok3233) I/hT2 [bli-4(e937) let-?(q782) qIs48] (I;III)* | 20 | *aak-2 RNAi* | 21 | 44 | -8.7% | 0.5742 |
| XVII | N2 | *-* | 20 | *hsf-1 RNAi* | 17 | 137 | - |  |
| XVII | VC2511 | *Y52B11A.2(ok3233) I/hT2 [bli-4(e937) let-?(q782) qIs48] (I;III)* | 20 | *hsf-1 RNAi* | 20 | 42 | 17.65% | < 0.0001 |
| XVIII | N2 | *-* | 20 | *L4440* | 24 | 192 | - |  |
| XVIII | VC2511 | *Y52B11A.2(ok3233) I/hT2 [bli-4(e937) let-?(q782) qIs48] (I;III)* | 20 | *L4440* | 28 | 84 | 16.67% | < 0.0001 |
| XVIII | N2 | *-* | 20 | *skn-1 RNAi* | 21 | 158 | - |  |
| XVIII | VC2511 | *Y52B11A.2(ok3233) I/hT2 [bli-4(e937) let-?(q782) qIs48] (I;III)* | 20 | *skn-1 RNAi* | 24 | 128 | 14.29% | < 0.0001 |
| XVIII | N2 | *-* | 20 | *atf-5 RNAi* | 24 | 142 | - |  |
| XVIII | VC2511 | *Y52B11A.2(ok3233) I/hT2 [bli-4(e937) let-?(q782) qIs48] (I;III)* | 20 | *atf-5 RNAi* | 24 | 100 | 0% | 0.0002 |
| XIX | N2 | *-* | 20 | *L4440* | 22 | 119 | - |  |
| XIX | VC2511 | *Y52B11A.2(ok3233) I/hT2 [bli-4(e937) let-?(q782) qIs48] (I;III)* | 20 | *L4440* | 24 | 108 | 9.09% | < 0.0001 |
| XIX | N2 | *-* | 20 | *skn-1 RNAi* | 21 | 105 | - |  |
| XIX | VC2511 | *Y52B11A.2(ok3233) I/hT2 [bli-4(e937) let-?(q782) qIs48] (I;III)* | 20 | *skn-1 RNAi* | 22 | 95 | 4.76% | 0.0744 |
| XIX | N2 | *-* | 20 | *atf-5 RNAi* | 22 | 110 | - |  |
| XIX | VC2511 | *Y52B11A.2(ok3233) I/hT2 [bli-4(e937) let-?(q782) qIs48] (I;III)* | 20 | *atf-5 RNAi* | 24 | 99 | 9.09% | < 0.0001 |
| XX | N2 | *-* | 20 | *L4440 - Larva* | 22 | 74 | - |  |
| XX | VC2511 | *Y52B11A.2(ok3233) I/hT2 [bli-4(e937) let-?(q782) qIs48] (I;III)* | 20 | *L4440 - Larva* | 23 | 94 | 4.55% | 0.0448 |
| XX | N2 | *-* | 20 | *skn-1 RNAi - Larva* | 16 | 60 | - |  |
| XX | VC2511 | *Y52B11A.2(ok3233) I/hT2 [bli-4(e937) let-?(q782) qIs48] (I;III)* | 20 | *skn-1 RNAi - Larva* | 12 | 60 | -25% | < 0.0001 |
| XXI | N2 | *-* | 20 | *L4440 - Larva* | 23 | 94 | - |  |
| XXI | VC2511 | *Y52B11A.2(ok3233) I/hT2 [bli-4(e937) let-?(q782) qIs48] (I;III)* | 20 | *L4440 - Larva* | 23 | 89 | 0% | 0.1358 |
| XXI | N2 | *-* | 20 | *skn-1 RNAi - Larva* | 16 | 76 | - |  |
| XXI | VC2511 | *Y52B11A.2(ok3233) I/hT2 [bli-4(e937) let-?(q782) qIs48] (I;III)* | 20 | *skn-1 RNAi - Larva* | 14 | 85 | -12.5% | 0.0002 |
| XXII | N2 | *-* | 20 | *L4440 - Larva* | 21 | 110 | - |  |
| XXII | VC2511 | *Y52B11A.2(ok3233) I/hT2 [bli-4(e937) let-?(q782) qIs48] (I;III)* | 20 | *L4440 - Larva* | 21 | 83 | 0% | 0.013 |
| XXII | N2 | *-* | 20 | *skn-1 RNAi - Larva* | 16 | 90 | - |  |
| XXII | VC2511 | *Y52B11A.2(ok3233) I/hT2 [bli-4(e937) let-?(q782) qIs48] (I;III)* | 20 | *skn-1 RNAi - Larva* | 14 | 33 | -12.5% | 0.1146 |
| XXIII | VP303 | *[nhx-2p::rde-1 + rol-6(su1006)]* | 20 | *L4440* | 18 | 111 | - |  |
| XXIII | VP303 | *[nhx-2p::rde-1 + rol-6(su1006)]* | 20 | *impt-1 RNAi* | 20 | 114 | 11.11% | 0.0402 |
| XXIV | VP303 | *[nhx-2p::rde-1 + rol-6(su1006)]* | 20 | *L4440* | 19 | 139 | - |  |
| XXIV | VP303 | *[nhx-2p::rde-1 + rol-6(su1006)]* | 20 | *impt-1 RNAi* | 12 | 156 | -36.84% | < 0.0001 |
| XXV | VP303 | *[nhx-2p::rde-1 + rol-6(su1006)]* | 20 | *L4440* | 18 | 85 | - |  |
| XXV | VP303 | *[nhx-2p::rde-1 + rol-6(su1006)]* | 20 | *impt-1 RNAi* | 13 | 157 | -27.78% | < 0.0001 |
| XXVI | NR350 | *[pDM#715(hlh-1p::rde-1) + pTG95(sur-5p::NLS::GFP)]* | 20 | *L4440* | 13 | 123 | - |  |
| XXVI | NR350 | *[pDM#715(hlh-1p::rde-1) + pTG95(sur-5p::NLS::GFP)]* | 20 | *impt-1 RNAi* | 13 | 84 | 0% | 0.0008 |
| XXVII | NR350 | *[pDM#715(hlh-1p::rde-1) + pTG95(sur-5p::NLS::GFP)]* | 20 | *L4440* | 12 | 39 | - |  |
| XXVII | NR350 | *[pDM#715(hlh-1p::rde-1) + pTG95(sur-5p::NLS::GFP)]* | 20 | *impt-1 RNAi* | 12 | 67 | 0% | 0.7766 |
| XXVIII | NR350 | *[pDM#715(hlh-1p::rde-1) + pTG95(sur-5p::NLS::GFP)]* | 20 | *L4440* | 16 | 27 | - |  |
| XXVIII | NR350 | *[pDM#715(hlh-1p::rde-1) + pTG95(sur-5p::NLS::GFP)]* | 20 | *impt-1 RNAi* | 16 | 33 | 0% | 0.6191 |
| XXIX | TU3401 | *[pCFJ90(myo-2p::mCherry) + unc-119p::sid-1]* | 20 | *L4440* | 20 | 64 | - |  |
| XXIX | TU3401 | *[pCFJ90(myo-2p::mCherry) + unc-119p::sid-1]* | 20 | *impt-1 RNAi* | 19 | 35 | -5% | 0.3699 |
| XXX | TU3401 | *[pCFJ90(myo-2p::mCherry) + unc-119p::sid-1]* | 20 | *L4440* | 17 | 64 | - |  |
| XXX | TU3401 | *[pCFJ90(myo-2p::mCherry) + unc-119p::sid-1]* | 20 | *impt-1 RNAi* | 17 | 79 | 0% | 0.2406 |
| XXXI | TU3401 | *[pCFJ90(myo-2p::mCherry) + unc-119p::sid-1]* | 20 | *L4440* | 17 | 34 | - |  |
| XXXI | TU3401 | *[pCFJ90(myo-2p::mCherry) + unc-119p::sid-1]* | 20 | *impt-1 RNAi* | 14 | 94 | -17.65% | 0.0017 |
| XXXII | N2 | *-* | 28 | *-* | 9 | 128 | - | - |
| XXXII | VC2511 | *Y52B11A.2(ok3233) I/hT2 [bli-4(e937) let-?(q782) qIs48] (I;III)* | 28 | *-* | 11 | 155 | 22.22% | 0.0001 |
| XXXIII | N2 | *-* | 28 | *-* | 9 | 97 | - | - |
| XXXIII | VC2511 | *Y52B11A.2(ok3233) I/hT2 [bli-4(e937) let-?(q782) qIs48] (I;III)* | 28 | *-* | 10 | 91 | 11.11% | 0.0001 |
| XXXIV | N2 | *-* | 28 | *-* | 9 | 128 | - | - |
| XXXIV | VC2511 | *Y52B11A.2(ok3233) I/hT2 [bli-4(e937) let-?(q782) qIs48] (I;III)* | 28 | *-* | 11 | 155 | 22.22% | <0.0001 |
| XXXV | N2 | *-* | 28 | *L4440* | 12 | 129 | - | - |
| XXXV | N2 | *-* | 28 | *impt-1 RNAi* | 13 | 119 | 8.33% | < 0.0001 |
| XXXVI | N2 | *-* | 20 | L4440 | 18 | 121 | - | - |
| XXXVI | N2 | *-* | 20 | *krs-1 RNAi* | 22 | 119 | 22.22% | < 0.0001 |
| XXXVI | ST60 | *gcn-1 (nc40) III* | 20 | *L4440* | 15 | 128 | - | - |
| XXXVI | ST60 | *gcn-1 (nc40) III* | 20 | *krs-1 RNAi* | 15 | 155 | 0% | 0.9226 |
| XXXVII | ST60 | *gcn-1 (nc40) III* | 20 | *L4440* | 15 | 99 | - | - |
| XXXVII | ST60 | *gcn-1 (nc40) III* | 20 | *krs-1 RNAi* | 15 | 108 | 0% | 0.6851 |
